# A systematic review of the use of topic models for short text social media analysis

**DOI:** 10.1007/s10462-023-10471-x

**Published:** 2023-05-01

**Authors:** Caitlin Doogan Poet Laureate, Wray Buntine, Henry Linger

**Affiliations:** 1 Faculty of IT, Monash University, Wellington Rd, Clayton, VIC 3800 Australia; 2 College of Engineering and Computer Science, VinUniversity, Vinhomes Ocean Park, Gia Lam District, Hanoi 10000 Vietnam

**Keywords:** Topic model, Social media, Short text, Twitter, NLP, LDA

## Abstract

Recently, research on short text topic models has addressed the challenges of social media datasets. These models are typically evaluated using automated measures. However, recent work suggests that these evaluation measures do not inform whether the topics produced can yield meaningful insights for those examining social media data. Efforts to address this issue, including gauging the alignment between automated and human evaluation tasks, are hampered by a lack of knowledge about how researchers use topic models. Further problems could arise if researchers do not construct topic models optimally or use them in a way that exceeds the models’ limitations. These scenarios threaten the validity of topic model development and the insights produced by researchers employing topic modelling as a methodology. However, there is currently a lack of information about how and why topic models are used in applied research. As such, we performed a systematic literature review of 189 articles where topic modelling was used for social media analysis to understand how and why topic models are used for social media analysis. Our results suggest that the development of topic models is not aligned with the needs of those who use them for social media analysis. We have found that researchers use topic models sub-optimally. There is a lack of methodological support for researchers to build and interpret topics. We offer a set of recommendations for topic model researchers to address these problems and bridge the gap between development and applied research on short text topic models.

**Supplementary Information:**

The online version contains supplementary material available at 10.1007/s10462-023-10471-x.

## Introduction and motivations

Social media disrupted the cultural, media and political landscape in new and unexpected ways, bringing with it new and interesting research opportunities to study social phenomena. However, social media is dynamic and both its form and effect. Societal norms, consumer behaviour, journalistic practices and media organisational strategies are rapidly evolving within these complex virtual environments.

As the online and offline world become further intertwined, researchers require new ways to study online social phenomena concerning offline situational contexts. Given that traditional data collection and analysis methods are unable to scale to meet the demands of social media data (SMD), these researchers have turned to computational methods to collect and analyse this data. One of these methods, topic modelling, has become popular with researchers looking to leverage SMD to study a phenomenon of interest (Rana et al. 2016; Abd-Alrazaq et al. 2020). Topic modelling of SMD has been conducted in many fields including journalism (Jacobi et al. 2016), public health (Han et al. 2020), urban planning (Haghighi et al. 2018), political science (Bail et al. 2018), and information systems (Pousti et al. 2021) to name just a few.

The increased interest of researchers^1^ in using topic modelling for social media analysis has motivated developers of topic models to extend the capabilities of these models for use on real-world SMD. In the last two decades, the nature of user-generated content has changed from longer message board posts and blog-style journals to shorter microblog posts created on platforms such as Twitter, Sina Weibo (Weibo), and Instagram. The brevity of microblogs is typically a result of a character limit imposed by the platform. For instance, Twitter has a limit of 240 characters (Rosen and Ihara 2017). However, SMD collected from platforms such as Twitter is more challenging to model. While earlier topic models such as the latent dirichlet allocation (LDA) (Blei et al. 2003) are capable of handling longer online content, they do not perform as well at generating semantic meaning from shorter texts (Yan et al. 2013; Mazarura and De Waal 2016; Zou and Song 2016). Consequently, short text topic model development continues to be an active area of interest in natural language processing (NLP) research.

**Fig. 1:**
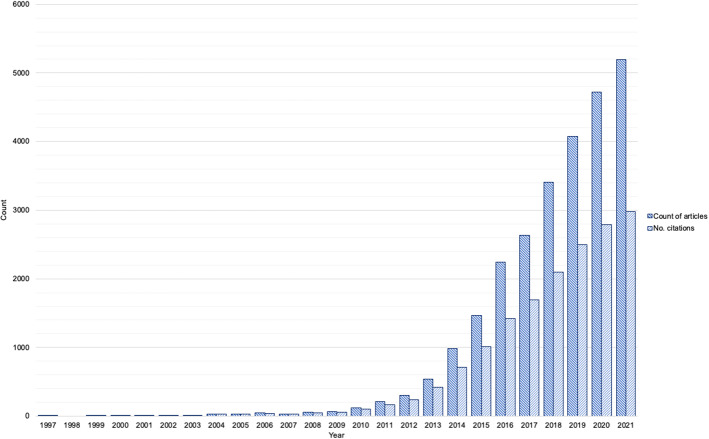
The number of topic modelling papers between 1997–2021 in venues and journals concerned with computer science (Dark blue). The number of citations per year of all topic modelling papers (light blue). The search string TI = (topic model*) was used to query results using WoS. These were restricted to document types: Article, Meeting (conference papers) and early access. The results were further restricted to those published in Computer Science journals. There were 2,604 articles returned. A citation analysis was conducted. The sum of times these articles were cited was 26,741 from 17,529 citing articles, and 22,501 without self-citation from 16,104 articles. The average citation per item was 10.27 citations. (Color figure online)

Topic modelling continues to be an active area of interest.. As shown in Fig. 1, the number of topic modelling articles published in computer science venues and journals each year is increasing at an exponential rate. Much of the focus of contemporary topic modelling research has been on overcoming challenges such as the data sparsity problem inherent to short texts (Tommasel and Godoy 2018; Albalawi et al. 2020). In recent years, there has been an influx of high-performance models (Zhao et al. 2021a), diversification of approaches (Zhao et al. 2019; Nugroho et al. 2020), and attention to evaluation and validation methods to empirically demonstrate superior performance when used on short text data (Bhatia et al. 2018; Hoyle et al. 2020; Doogan and Buntine 2021). Recent approaches to modelling short text datasets include the use of auxiliary metadata (Zhao et al. 2017), using contextual word embeddings (Huang et al. 2020) semantic anchors Steuber et al. (2020), application of neural approaches (Zhao et al. 2021a) attention to the issue of heavily imbalanced datasets (Zuo et al. 2016), and neural approaches (Wu et al. 2020b; Zhao et al. 2021b).

The application of topic modelling for social media analysis has been well established in the scientific literature (Jacobi et al. 2016; Curiskis et al. 2019). However, there is a growing concern that topic modelling development is becoming disconnected from the application of these techniques in practice (Lee et al. 2017; Hoyle et al. 2020; Doogan and Buntine 2021). NLP researchers have begun to consider whether topic modelling is sufficiently robust for applied research on real-world problems. For example, Bose et al. (2021) reports that despite the promise of cross-domain generalisability, sophisticated topic models perform poorly in hate-speech detection tasks. Recent re-evaluation of existing topic models have yielded results that contradict the original research articles (Mazarura and De Waal 2016; Harrando et al. 2021), revealed problematic methodological practices (Doogan and Buntine 2021), cast doubt over the rigour of standard research frameworks (Lau et al. 2014; Hoyle et al. 2020; Doogan and Buntine 2021), and raised epistemological questions concerning the utility of topic models (Nguyen et al. 2020).

Several surveys have been conducted on topic modelling as shown in Table 1. However, few of these surveys focus on short texts and social media (Nugroho et al. 2020; Qiang et al. 2020). While these surveys provide some insight into applications of topic models Hannigan et al. (2019), they do not offer an in-depth understanding of how and why topic models are used for applied research that uses SMD.

**Table 1:** Surveys on topic modelling in order of latest to earliest publication year

Article	Years	Description	No. Articles
Zhao et al. (2021a)	2009–2020	A survey of recent developments in neural topic models (NTM) concluding with a summary of three sets of challenges and opportunities of NTMs are provided.	74
Chauhan and Shah (2021)	1986–2020	A review and experiment to compare the main topic model classes included LDA-extensions, extensions, hierarchical, word embedded and multilingual models. Evaluation and implementation techniques are covered.	185
Qiang et al. (2020)	1989–2019	Offers a taxonomy of algorithms for short text topic modelling. Defines different modelling tasks and summaries the challenges and future directions for the field.	74
Nugroho et al. (2020)	1990–2019	A survey of the approaches for Twitter data topic modelling. Models are discussed in terms of their feature input, evaluation and applications.	137
Vayansky and Kumar (2020)	1983–2018	Presents an analysis of non-LDA based methods and provides a decision tree of to determine which topic modelling methods are best for a given analysis.	35
Jelodar et al. (2019)	2003–2016	Topic model research development, applications, and trends. Specifically, those which are extensions of LDA.	158
Xia et al. (2019)	1983–2019	A survey of three categories of topic modelling methods for text classification and summary of their advantages and limitations.	38
Likhitha et al. (2019)	1998–2019	An overview of topic modelling evolution and extraction methodologies for short texts. Provides a comprehensive inventory of benchmark datasets for short texts.	84
Mulunda et al. (2018)	1984–2018	Provides a classification and summary of techniques, tools and inference algorithms for topic models and a brief overview of applications.	85
Liu and Tang (2018)	2003–2018	A summary of the multi-label topic modelling literature. The literature is categorised into four model types by the authors.	15
Zhou et al. (2017)	2007–2017	A review of three topic evolution models (discrete time, continuous time, and online topic model) and their applications.	43
Kjellin and Liu (2016)	1990–2015	Surveys literature on and identifies trends on, interactivity and visualisation of topic models with a focus on manual (human) interpretation.	26
Chen et al. (2016)	1999–2014	A review of the application of topic modelling to software engineering to provide visibility for topic modelling development and insights for software engineers.	167
Sun et al. (2016)	2003–2015	Topic model applications to software engineering and development tasks.	38
Rana et al. (2016)	2010–2016	Review and comparison of LDA-based topic modelling techniques for sentiment analysis.	16
Alghamdi and Alfalqi (2015)	2001–2011	Classifies and reviews prominent topic models in two major topic modelling categories: Topic modelling methods and topic evolution methods.	23

There is little visibility over the use of topic models and whether they are adequately meeting the needs of the researchers who employ them (Lee et al. 2017). A lack of knowledge about why, how and who is using topic models for social media research is problematic for two reasons. First, topic modelling developers may not be aware of instances in which topic models fail to perform as promised. There is a possibility that the use of models that perform sub-optimally continues unchecked. The second reason is that a lack of visibility will result in missed opportunities to optimise topic model performance in future research strategically.

This research aims to determine who uses topic models for social media analysis, why, and how they are using them. Additionally, we analyse this literature and draw on the author-identified limitations and opportunities to develop a set of recommendations for topic modelling researchers for future work on topic models for social media datasets. To achieve this, we have conducted a systematic literature review (SLR) of 189 recent articles that apply topic modelling to short text SMD, including a critical analysis of 99 of these articles.

The methodological contribution of this research is to the broadening debate about scientific rigour in NLP, such as the importance of user-orientated research directions, contribution to model development and topic model evaluation. By identifying the benefits and pitfalls that may exist for those using these tools, we can provide a basis to improve the use of these models by applied researchers to analyse social media data. This methodological contribution has been derived from a synthesis of the literature resulting in a set of recommendations for developers covering three dimensions—Approaches, user knowledge, and research advancement. Guidance on approaches encourages developers to become familiar with the aims of the user and the methodologies into which they are building topic modelling. A key recommendation is to adopt an application-driven design where utility is demonstrated by case studies informed by subject matter experts. Recommendations focused on user knowledge aim to bridge the research (and knowledge) gap between empirical and applied works to reduce the amount of ’guess work’ users undertake. Practical steps that, if taken, will support this aim are highlighted and include increased transparency about experimental settings, basing development on application needs rather than just ML problems, and engaging with the applied literature. The final set of recommendations addresses how developers can support research advancements in ML and those disciplines in which topic models are used. For instance, there is a pressing need for user-friendly tools and software that provide state-of-the-art approaches. Popular packages have reported limitations that will negatively affect results in applied studies. A critical recommendation is to improve the validity of topic modelling evaluation and align these measures with the needs of users. Moreover, the findings from this study will address the question of how to assess the established methodological robustness of topic modelling research.

## Methodology

A SLR is a rigorous and practical approach to establishing the volume, significance, consistency and relevance of a specific selection of peer-reviewed literature, ensuring objective, accurate and reliable conclusions (Tranfield et al. 2003). By adopting a SLR methodology, relevant studies about applied topic modelling for social media are reviewed, critically appraised and synthesised to provide the means to integrate practical experience with the best evidence from the research into the decision making process regarding the development and use of topic modelling for social media (Kitchenham et al. 2009, 2004).

Additionally, the rigour of an SLR methodology strengthens the legitimacy and authority of the evidence from which this guidance is formulated. This SLR has drawn on the methodologies described by both Colicchia and Strozzi (2012) and Denyer and Tranfield (2009) to produce a transparent, objective and heuristic account of the recent research conducted. These qualities are critical to achieving the aims of this study.

### Databases and search terms

Articles were collected through searches of the Ebsco^®^ and Web of Science^®^ (WoS) literature databases. These databases were chosen based on their broad coverage of research subjects (Rashman et al. 2009). The search was restricted to peer-reviewed journal articles and conference publications written in English. Keywords were queried as a set of terms or a combination of terms with Boolean operators. For example, [“topic model*” AND “*social media*” OR “*twitter*” OR “*instagram*” OR “*reddit*”]. These search strings and keywords (including the social media platforms queried) are listed in Appendix 6. While the focus of the SLR is on short text topic modelling, social media platforms that allow for the creation of longer posts, such as *Facebook* and *LinkedIn* have been included in search strategy as posts on these platforms are typically far shorter than these character limits. The search was restricted to articles published between January 2016 and June 2021, capturing articles across a period of 5.5 years. A total of 1284 articles were retrieved.

Journal articles were restricted to those published in high-quality journals determined according to the SJR^2^ and SNIP.^3^ quality measures. Specifically, this SLR accepted only those articles that were ranked in the top 25% of journals in at least one subject category informed by Scopus^®^, or that obtained a 2020 SNIP of 1.5 or higher. Conference papers were restricted to those that were ranked in CORE 2020 as A or $\hbox {A}_{*}$.^4^ Once articles that did not meet the quality criteria and all duplicates were removed, 546 publications remained.

### Exclusion criteria

This SLR sought to identify those articles where topic modelling was employed to investigate some phenomenon. Research that did not fit this description was excluded from this study. The complete list of exclusion and inclusion criteria are described in Table 2. Due to the large number of articles returned from the keyword searches, and the need to reduce duplicates between these, the eligibility criteria were not applied until the screening process had begun. Among these was the exclusion of articles that presented a new topic modelling method or procedure, including the small number that evaluated the work on SMD through ‘case studies’. This was because the primary aim of the paper was to introduce a new method and not to yield insights from SMD that would inform the study of some phenomena of interest. Moreover, articles that introduce new methods and processes are typically authored by those with technical expertise in NLP. The restrictions for searching and curating articles, inclusion, and exclusion criteria applied to the collection is shown in Table 2.

**Table 2:** Search restrictions, and the inclusion and exclusion criteria for screening articles

Search restriction	Inclusion criteria	Exclusion criteria
Q1 or SNIP < 1.5	SMD is modelled	SMD is not modelled
CORE ranking of A or A*	Topic modelling is a core method	Topic modelling is not a core method
Published 2016–2021	Investigates a real world phenomenon	New model or process
English language	Analysis of topics is conducted	Analysis of topics is not reported
Peer-reviewed	Interpretation is discussed.	Does not provide insights from topics

### Screening process

**Fig. 2:**
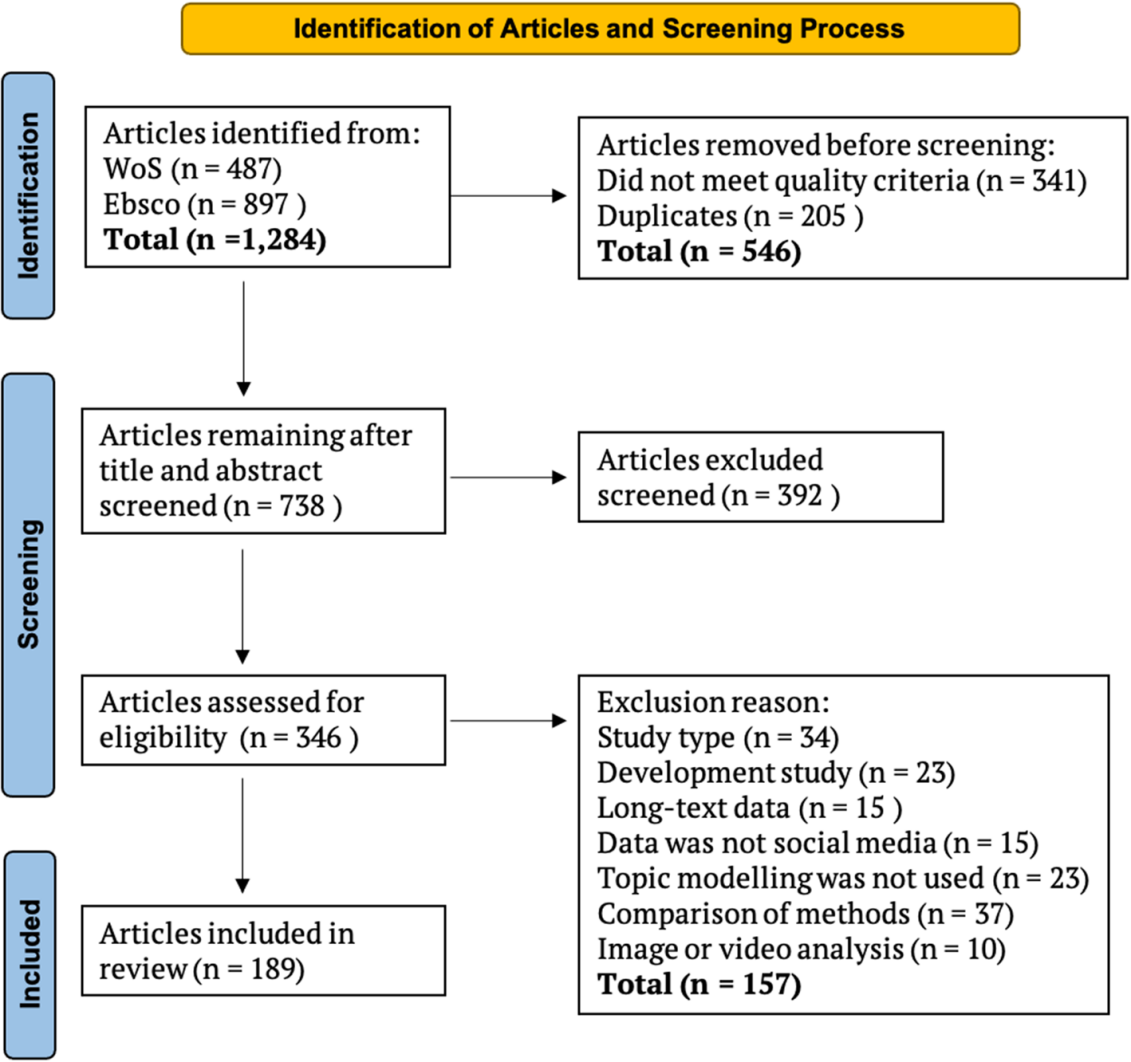
PRISMA style flow chart detailing the collection and article screening process

The screening process was conducted in two stages. All 738 titles and abstracts were read and evaluated against the inclusion criteria (Tranfield et al. 2003). Following this, 346 articles were read in full, and articles that did not meet the eligibility criteria were excluded. This process resulted in 189 articles being included in this SLR. Figure 2 details the screening processes as demonstrated by (Lima et al. 2021) in their adaption of the PRISMA framework presented by (Moher et al. 2010).

### Analysis

All articles were uploaded into the online platform Covidence^5^ for data extraction. Data extraction tables were constructed and included elements such as the area of study, motivations, data preparation, topic modelling procedure, evaluation and interpretation. The data extraction tables were also used to capture descriptive data, including publication year, discipline and research area^6^, the rationale for using topic modelling, models used and from which platform the data was retrieved. The data extraction template is available in Appendix 6.

Each article was classified into a disciplinary category and research area to determine which disciplines adopt topic modelling (See Appendix 6 for an explanation of this categorisation). In total, 44 final research categories were assigned. These categories were further aggregated into 17 categories and grouped under their research area according to the WoS schema.^7^.

The analysis of this literature was conducted in two stages. In the first stage, all 189 articles were read, and the data necessary to map out the existing literature was extracted. In doing this, we could refine our existing line of enquiry further. Following this, we conducted a fine-grained analysis of 99 articles. We did not pursue a review of all articles as the trends in the data extraction remained stable, indicating that saturation had been reached and no new knowledge would be gained from a complete review (Booth 2001). This process allowed us to synthesise ‘best evidence’ to provide insights and guidance for practitioners and scientists working on and with topic models.

## Results

### Research areas and disciplines

**Fig. 3:**
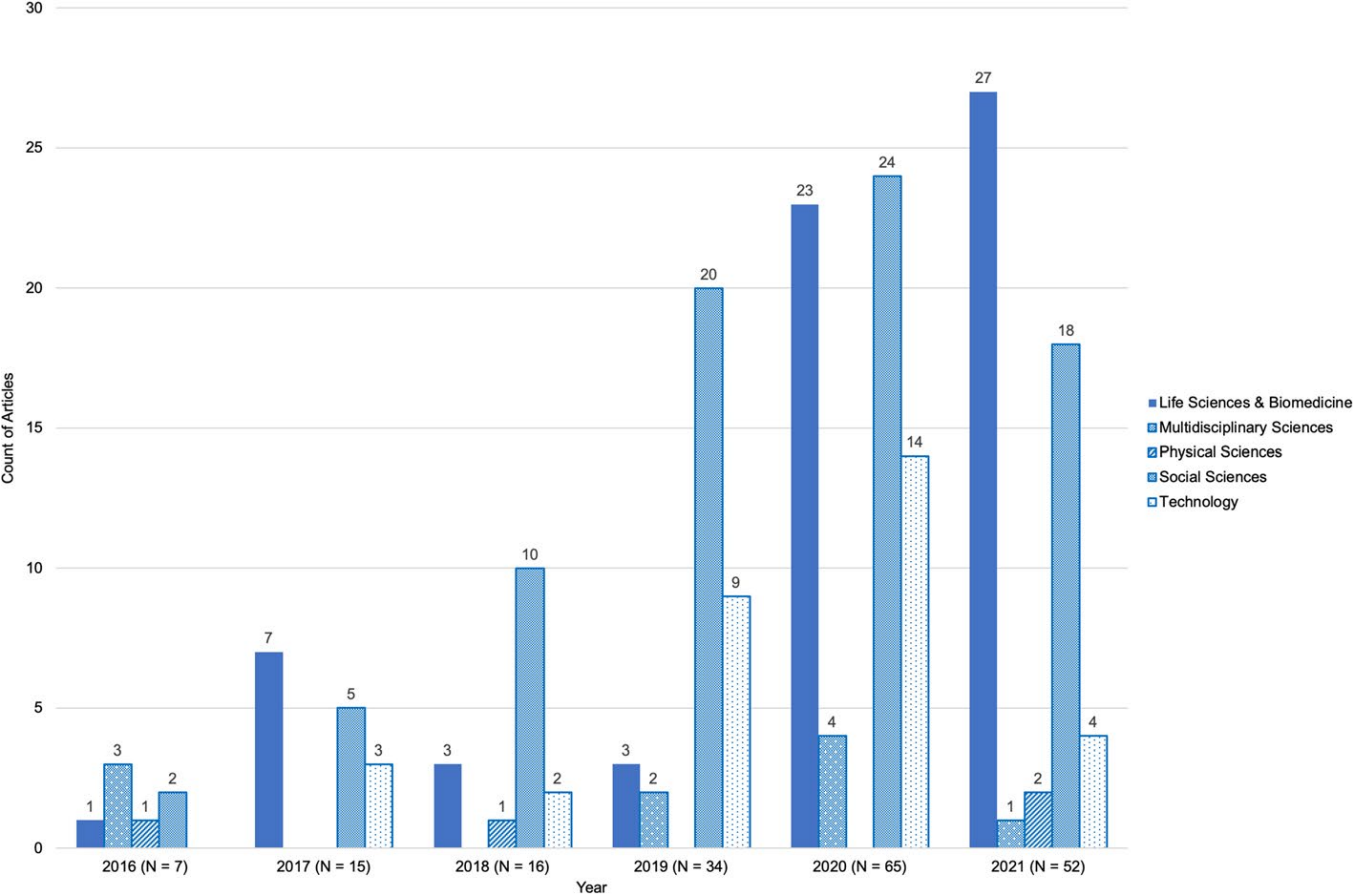
The number of articles published each year from 2016 to 2021 ($n = 189$) for each research area

During the period studied, publications have climbed dramatically, from 7 in 2016 to 65 in 2020. Given that 52 papers were published in the shortened 2021 six-month collection period, as shown in Fig. 3.

**Fig. 4:**
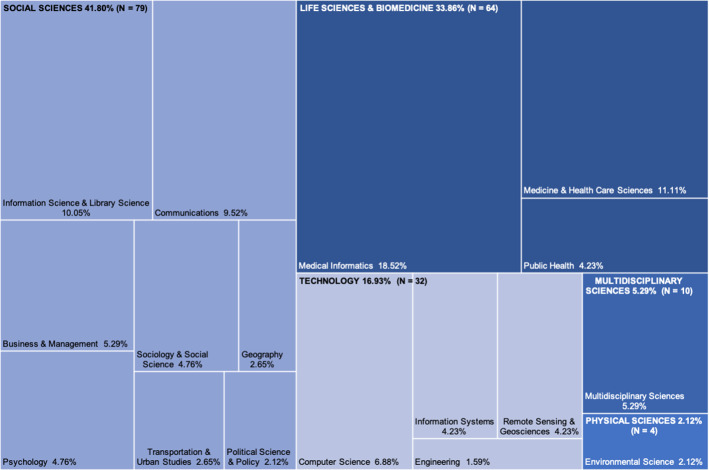
Proportion of disciplinary studies of each research area in the collection that used topic modelling for SMD ($n = 189$)

SMD studies that employ topic modelling are conducted throughout a range of research areas and disciplines (See Fig. 4). A sizeable proportion of these works (41.80%, $n = 79$) are assigned to the Social Sciences research area. Eight disciplines were found from articles in this analysis. Within this research area, the greatest number of articles were published in Information Science & Library Science disciplinary journals (24.05%, $n = 19/79$), followed by Communications journals (22.78%, $n = 18/79$). These two disciplines account for 10.05% and 9.52%, respectively, of all articles in the collection. The research area of Life Sciences and Biomedicine was less diversified, with three disciplines contributing 33.86% ($n = 64$) of all articles. Within this research area, 54.69% ($n = 35$) of articles are published in Medical Informatics journals, while 32.81% ($n = 21$) are from the Medicine and Health Care Sciences. These two disciplines are represented in 18.52% ($n = 35/189$) and 11.11% ($n = 21$) of all articles in the collection, respectively.

Although the two most prominent research areas were Social Sciences and Life Sciences and Biomedicine, in the last 18 months of the collection period (January 2020 to June 2021), the number of articles published in the Life Sciences and Biomedicine surpassed those published in the Social Sciences (See Fig. 3). The most prominent discipline in the collection was Medical Informatics with JMIR the most popular publication venue, an early adopter of topic modelling applications. Many of the papers published in JMIR (62.07%, $n = 18(29)$) focused on the COVID-19 pandemic and used an exploratory strategy known as Infodemiology, which was popularised in JMIR (Eysenbach et al. 2009).

COVID-19 related studies made up 35.04% ($n= 41$) of all articles published between 2020 and 2021 ($n = 117$) across all venues, with 73.2% coming from Life Sciences and Biomedicine disciplines ($n = 30$). Medical informatics journals published the most COVID-19 studies ($n = 27$). Research concerning the COVID-19 Pandemic has likely been a catalyst for the growth in social media analysis in medical informatics studies as research was constrained by restrictions on researcher interaction with participants in many countries. Social media was an attractive data source as it was accessible (Cuello-Garcia et al. 2020) and a rich source of data regarding life during the pandemic as lockdowns and stay at home orders drove online social interaction (Wong et al. 2021).

### Journals

An analysis of the 111 journals represented in the collection showed that the journal of medical internet research (JMIR) published 29 articles (15.34%) followed by nine articles in PLoS One (4.76%), and five (2.65%) each in the International Journal of Disaster Risk Reduction and International Journal of Information Management. The journals with the most articles published are listed in Table 3 (for a complete list, see Appendix 6).

**Table 3:** Journals with the largest number of articles in the collection

Journal	Research category	Count	Prop.
J. Med. Internet Res.	Medical informatics	29	15.34%
PLOS ONE	Multidisciplinary sciences	9	4.76%
Int. J. Disaster Risk Reduct.	Remote sensing and geosciences	5	2.65%
Int. J. Inf. Manage.	Information science and library science	5	2.65%
IEEE Access	Computer science	4	2.12%
Inf. Process. Manage.	Computer science	4	2.12%
Info. Commun. Soc.	Information systems	4	2.12%
Online Inf. Rev.	Information science and library science	4	2.12%

### Applications and approaches

Most studies used topic modelling to isolate manageable collections of semantically similar documents. Studies adopting a case study approach treat topics as artefacts of social media discourse that are anchored in the real world. These studies aim to draw inferences about a real-world environment based on the relationship between topics and other factors (Joo et al. 2020). For example, Liang et al. (2019) sought to determine if there was an association between information and social environments online to the regional prevalence of obesity. In other studies, researchers wanted to determine if topic models aid in predicting real-world events from social media posts. Kurten and Beullens (2021) wanted to know if the number of tweets differs as a function of the pandemic’s timeline and related steps and how the content of these tweets shifts over time.

Other studies were exploratory and descriptive, aiming to provide a broad overview of the topics associated with a specific group of people, an event, or some other social phenomenon. Nobles et al. (2020) used topic modelling to understand the way that those who self-identified as having HIV communicated their lived experience with the disease.

Topic modelling was also used to harness real-time communication signalling via social media platforms for disaster and crisis management communication, monitoring and response (Fischer-Preßler et al. 2019; Xu et al. 2019; Deng et al. 2020). While much of this work is theoretical, adopting a case study approach (Fischer-Preßler et al. 2019; Deng et al. 2020), studies such as Zhang et al. (2021) focus more on the development of frameworks that employ topic models to construct signals from SMD and geographic information to provide information about different disaster events.

Most studies used more than one computational technique. Topic modelling was used to conduct a content analysis in combination with other methods such as sentiment analysis or network analysis (Ibrahim and Wang 2019b). Additional approaches were either deployed on topic-specific document collections (Zhu et al. 2020; Xue et al. 2020b), or in addition to the topic modelling content analysis (Liu 2020). Topics were also used as input features for other computational or statistical approach. In their study of radicalised online content, Abdul-Rahman et al. (2021) used a feature enrichment approach to model topics from tweets. The topics were used to classify actors into Pro-ISIS and Anti-ISIS categories. The numerous studies concerning the COVID-19 global pandemic (Doogan and Buntine 2021; Kurten and Beullens 2021) were directed to providing information to policymakers and healthcare organisations to address the needs of stakeholders (Abd-Alrazaq et al. 2020). A number of these studies adopted an Infodemiology (i.e., information epidemiology) or Infoveillance approach, particularly in Health Informatics articles (Xue et al. 2020b, 2020c; Medford et al. 2020)

There were various interpretations of topic modelling regarding its status as a methodology, a computational method, or an automated tool. Few studies specified what topic modelling was other than to provide a brief description of the modelling process. Several studies provided a structured, sequential process for conducting topic modelling and made claims of a novel framework. Of interest was that these frameworks were more or less the same, despite being developed within specific disciplines, including transportation and urban studies (Abdul-Rahman et al. 2021), operations research (Ibrahim and Wang 2019b), and emergency management (Wu et al. 2020a). One reason for the similarity of frameworks could be that these studies were the first demonstration of the capabilities of topic modelling for social media analysis in their discipline, often drawing on the same foundational papers (Al-Ramahi et al. 2017; Chae 2019; Puschmann et al. 2020; Gregoriades and Pampaka 2020).

A small number of studies included topic modelling as part of their mixed-methods approach. In these studies, topic modelling was positioned as a method alongside autoethnography (Brown 2019), grounded theory (Xu and Xiong 2020), regression analysis (Chan et al. 2020), and surveys (Lock and Pettit 2020; Svartzman et al. 2020). A case study approach was adopted in several studies to build or extend theoretical frameworks (Kwon et al. 2019; Zhang et al. 2020; Bérubé et al. 2020). A description of the approaches identified in this study is available in Appendix 6.

### Data sets

#### Sources

Fourteen social media platforms were identified as data sources. The majority of studies used only one data source ($n = 179$), nine used two sources, and one used three different sources (Nizzoli et al. 2020). Twitter was the most popular source, followed by Reddit, Sina Weibo, and Facebook. The social media platforms identified as data sources are shown in Table 4.

**Table 4:** Social media data sources

Data source	Description	Proportion (Count)
Twitter	Micro-blogging platforms where users share posts, photos and other media using hashtags and mentions of other users.	68.34% ($n = 136$)
Reddit	A collection of forums (subreddits) where users can share news and content, or comment on other users’s posts.	9.05% ($n = 18$)
Sina Weibo	Chinese micro-blogging platform where users share posts within their network.	8.04% ($n = 16$)
Facebook	A social networking platform where users share posts, photos, and other media and can comment on those posts of people in their network.	7.04% ($n = 14$)
Instagram	Photo sharing social network platform. Posts are accompanied by descriptive text and hashtags.	2.01% ($n = 4$)
Yelp	Crowd-sourced reviews of businesses and restaurants.	1.01% ($n = 2$)
YouTube	Comments left on video uploads by users of the platform.	1.01% ($n = 2$)
Blued	A Chinese social networking app for gay men.	0.50% ($n = 1$)
Discord	A real-time Voice over IP (VoIP) platform targeted to gamers.	0.50% ($n = 1$)
Google (Reviews)	Google hosted crowd-sourced reviews and ratings for businesses and places of interest.	0.50% ($n = 1$)
Apple (Reviews)	Apple hosted crowdsourced reviews of products made available through the Apple app store.	0.50% ($n =1$)
Niche	Social networking platform that allows users to review neighbourhoods, schools, shops and other local amenities.	0.50% ($n = 1$)
Telegram	An encrypted instant messaging app that allows video calling, VoIP, and file sharing.	0.50% ($n = 1$)
Trip advisor	Crowd-sourced reviews of locations, tourist destinations, hotels and restaurants.	0.50% ($n = 1$)

#### Data preparation

Most studies reported the undertaking of data preprocessing, though only a few explained the methodological rationale that informed their choices (Bérubé et al. 2020; Svartzman et al. 2020; del Gobbo et al. 2021). A broad array of preprocessing tasks was observed, the most interesting trends seen across the various preprocessing tasks are discussed here.

##### Denoising

Removal of special characters was broadly conducted but was not consistent. Emails, URLs and HTML were commonly removed (Bahja and Safdar 2020; Chen et al. 2020; Feldhege et al. 2020), as were accents (Nolasco and Oliveira 2020). Denoising was generally performed well, but some studies also performed atypical procedures, potentially degrading topic model performance. For example, replacing characters with their word form such as ‘$’ to ‘dollar’ (Gregoriades and Pampaka 2020) and ‘#’ to ‘hashtag‘ before appending this word to the hashtag-word itself (Carlson and Harris 2020).

##### Normalisation

In studies where sentiment analysis was conducted on the same preprocessed dataset, replacement of special characters was only conducted where the word was not capitalised or punctuated (Reyes-Menendez et al. 2020). Similarly, while the majority of articles reported removing numbers, some replaced them with the written term (Zhou and Na 2019; Gregoriades and Pampaka 2020), which does not assist overly in curating the documents appropriately for modelling and would degrade the quality of topics. Other authors made decisions that were not explained, such as in (Zhai et al. 2020) where all punctuation marks were removed from the collection of tweets except for periods, semicolons, question marks, and exclamation marks. Punctuation was not removed in all studies (Jamison et al. 2020; Yu et al. 2021)

Similarly, few studies removed the keywords used to query the data. The removal of keywords is critical to ensure the quality of topics and ease of interpretation. For instance, Xu et al. (2019) collected tweets about a controversial 2019 marketing campaign run by the shaving product company Gillette using the hashtag #gillette. As they did not remove this hashtag or the word ‘Gillette’, every topic would likely begin with the query term. Some authors left keywords in to try and force the specification of topics (Carlson and Harris 2020). (Okon et al. 2020) appended the subreddits‘r/schizophrenia’, ‘r/SuicideWatch’, and ‘r/Depression’ to each comment (Low et al. 2020) to seed the differentiation of topics related to them in their study of dermatology patients. Although underreported in general, removal of keywords was most common in studies using tweets (19.12%).

Stopwords were removed in 86.90% of studies. Bespoke stopword lists were common in studies using tweets (Zheng and Shahin 2020; Wicke and Bolognesi 2020). Words included were either of a high frequency and would introduce noise (Jeong et al. 2019), or were specific to the domain and would bias topic formation (Valdez et al. 2020; Doogan et al. 2020). Only 18.20% of studies reported removing domain-specific words, and 21.20% reported removing low or high-frequency tokens.

##### Structural processing

Multi-lingual data management strategies were reported in 45.50% of studies. The first was only seen in studies using tweets were collection packages such as Twint (Doogan et al. 2020), and Twarc (Alshalan et al. 2020) can be tailored to retrieve tweets in a specific language such as Arabic (Alshalan et al. 2020), Spanish (Mostafa and Nebot 2020), German (Fischer-Preßler et al. 2019), or English (Medford et al. 2020; Pavlova and Berkers 2020). The second strategy was to filter out undesired documents from the collection using packages such as the Python packages LangID (Doogan et al. 2020; Nobles et al. 2020) and PolyGlot (Nizzoli et al. 2020). The third strategy was to translate the documents using the Google Translate API (Zhang et al. 2020; Peres et al. 2020), or Google’s Compact Language Detector packages (Feldhege et al. 2020). In the case of Chinese, Japanese, and Korean (CJK) languages, text segmentation of characters and morphological analysis was required before translation (Li et al. 2020b; Kitazawa and Hale 2021). The JiebraR package (Deng et al. 2020; Zhu et al. 2020; Li et al. 2020a; Wu et al. 2021) and ictclass (Wang et al. 2020) Python package were exclusively used for documents collected from the Chinese social media platform Sina Weibo.

The majority (62.5%) of authors failed to declare the approach taken to tokenisation. A further 56.57% did not evidence that stemming or lemmatisation was conducted^8^. Of those that did, 51.6% reported treating the documents through stemming, 37.21% through lemmatisation, and a further 11.63% through applying both techniques. A small number of studies (12.10%) reported conducting Parts-of-speech (POS) tagging either to enhance lemmatization (Liu 2019; Abd-Alrazaq et al. 2020) or to isolate nouns and adjectives before re-modelling (Kirilenko et al. 2021). Bigrams were generated for 20.20% of studies, though this did not appear to improve the interpretability of topics (Medford et al. 2020). @articleliu2020analyzing, title=Analyzing the impact of user-generated content on B2B Firms’ stock performance: Big data analysis with machine learning methods, author=Liu, Xia, journal=Industrial Marketing Management, volume=86, pages=30–39, year=2020, publisher=Elsevier

##### Document length

A small number of studies ($n = 12$) reported removing documents with a low number of tokens. This was either conducted before preprocessing (Chae 2019; Reyes-Menendez et al. 2020), or after preprocessing. The lower threshold was between 2 tokens (Wicke and Bolognesi 2020; Feldhege et al. 2020) and 10 tokens (Doogan et al. 2020; Vaughan 2020). An upper limit for document length was set in one study (Kirilenko et al. 2021), where documents collected from TripAdvisor reviews that were $> 4$ or $< 25$ tokens were excluded.

### Topic modelling

#### Topic models

Fifteen topic models were identified in the analysis. LDA (Blei et al. 2003) was used in 79.79% ($n = 154/189$) of studies. This is an interesting finding as it has been well documented that LDA is not optimal for short texts (Yan et al. 2013; Mazarura and De Waal 2016; Zou and Song 2016). The next most frequent model used was the Structural Topic Model (STM) implemented by Roberts et al. (2014), which was adopted in 13 studies (6.74%). All studies that used this version of STM were within the Social Science research area.

The majority of studies only used one topic model (*n* = 185), four studies made use of two topic models. In these studies, LDA was combined with either Dirichlet multinomial mixture (DMM) model (Yin and Wang 2014; Surian et al. 2016), dynamic topic model (DTM) (Blei and Lafferty 2006; del Gobbo et al. 2021), multi-grain topic model (MG-LDA) (Titov and McDonald 2008; Hu et al. 2019) or Biterm Topic Model (BTM) (Cheng et al. 2014; Pang et al. 2020) (Table 5).

**Table 5:** Topic models used for social media analysis ($n = 193$)

Topic model	Number of papers
Latent dirichlet allocation (LDA)	155
Structural topic model (STM)	13
BiTerm topic model (BTM)	9
Non-matrix factorization (NMF)	3
Dirichlet multinomial mixture (DMM)	2
Dynamic topic model (DTM)	2
Guided-LDA	1
Correlation explanation (CorEx)	1
Joint sentiment topic model (JST)	1
Labelled-LDA	1
Latent feature LDA (LF-LDA)	1
MetaLDA	1
Multi-grain topic model (MG-LDA)	1
Polylingual topic model (PTM)	1
Single topic LDA (ST-LDA)	1

#### Model optimisation and evaluation

The most common way authors were seen to decide on a value for *K* was to calculate one of several metrics traditionally used to empirically validate the performance of a topic model on benchmark datasets. Several studies made use of a perplexity curve (Al-Ramahi et al. 2017; Hwang et al. 2020; Thorson et al. 2020; Qi et al. 2020; Zhang et al. 2021), or a combination of perplexity and coherence scores (Hemmatian et al. 2019; Chan et al. 2020; Kirilenko et al. 2021). Several authors established perplexity but could not describe why it was being used to optimise *K* (Chan et al. 2020). A range of coherence scores were employed including *C*_*Umass *_(Hemmatian et al. 2019; Xue et al. 2020b; Pang et al. 2020; del Gobbo et al. 2021), *C*_*NPMI*_(Deng et al. 2020; Doogan et al. 2020; Hacker et al. 2020), *C*_*V*_(Murashka et al. 2020), *C*_*PMI*_ (Bahja and Safdar 2020). The majority of studies did not specify which coherence measure were used (Medford et al. 2020; Kirilenko et al. 2021). This is typical of studies using Gensim (Xue et al. 2020a, 2020c; Valdez et al. 2020) which offers several coherence measures. The authors also specifically stated that they aimed to produce topics with certain qualities, including interpretability, specificity, stability and exclusivity.

##### Interpretability

When reviewing topics, authors looked for qualities including interpretability (Jenkins et al. 2016; Meyer et al. 2019; Amin et al. 2020; Okon et al. 2020; Yu et al. 2021). An interpretable topic is one that intuitively makes sense and is easy to label. Manual analysis of topics was conducted in combination with evaluation measurements. The majority of authors reviewed only the top topic terms (Gurajala et al. 2019; Hemmatian et al. 2019; Hacker et al. 2020), although some authors included the most representative documents for their review of topics (Fischer-Preßler et al. 2019; Feldhege et al. 2020; Doogan et al. 2020). Coherence scores are an accepted proxy for interpretability.

##### Specificity

Studies seeking highly specific topics optimised using specificity measures (Nizzoli et al. 2020; Cesare et al. 2020) such as cosine similarity (Jeong et al. 2019; Chae 2019). A manual inspection for specificity was conducted by manual inspection of topics at each value of *K* (Xu and Zhou 2020; Peres et al. 2020; El-Bassel et al. 2021; ).

##### Stability

Authors also sought the persistence of topics as an indicator of the optimal value for *K*. Manual inspection of topics was conducted at different values of *K* (Brown 2019), as well as formal stability analyses (Greene et al. 2014). Topic stability across runs was used by Hemmatian et al. (2019).

##### Exclusivity

Exclusivity appears to be favoured by several authors as a sought after quality in topics (Li et al. 2020a; Kitazawa and Hale 2021). Often it was seen to be quantitatively measured and then supported by manual analysis to determine the degree of thematic commonality between topics (Kwon et al. 2019; Fischer-Preßler et al. 2019; Hacker et al. 2020). Others introduced novel measures, for example, topic concentration (Abd-Alrazaq et al. 2020).

### Software

The packages, programs, and tools that researchers used to preprocess data and implement topic models were analysed. The most common preprocessing tool identified by authors was the natural language tool kit (NLTK) (Loper and Bird 2002; Bird and Loper 2004).

Topic modelling was most frequently conducted using either Gensim (Řehůřek and Sojka 2010) in Python (31.30%) and/or MALLET (McCallum 2002) (26.87%). Three studies reported using Gensim as a wrapper for MALLET (Yan et al. 2020; Pavlova and Berkers 2020; Nobles et al. 2020). Aside from the different languages, Genism and MALLET implement different inference algorithms for LDA. Gensim implements an online variational Bayes algorithm (Hoffman et al. 2010), whereas MALLET uses an optimised Gibbs sampling algorithm (Yao et al. 2009). Aside from LDA, Gensim was used for both papers that used DTM (Ha et al. 2017; del Gobbo et al. 2021). MALLET was found to be used to implement LDA, MetaLDA (Doogan et al. 2020), and PTM (Pruss et al. 2019). Other notable tools were the stm package in R (Roberts et al. 2014).

## Discussion

A review of the limitations and opportunities for using topic models, as stated in the reviewed studies, has provided insights into what researchers need from topic modelling and the implications of these needs for topic modelling developers. This section summarises the limitations of topic modelling as directly stated by the authors of the reviewed studies and the opportunities that topic modelling presents for applied research. The limitations and opportunities have been grouped into three distinct categories for discussion. These categories are approaches, user knowledge, and research advancement. We comment on the implications of these findings for future topic model development research and contribute a number of recommendations for those developing topic models. These recommendations may assist in improving the validity, usability and usefulness of topic models for applied research using SMD.

### Approaches

Topic modelling is a novel technique for applied researchers which has only recently gained traction across a variety of disciplines (See Fig. 3). There is no standard approach to topic modelling and interpretation in the applied literature. Several authors identified their use of topic modelling as an opportunity as it exposed other researchers in their fields to topic modelling and demonstrated the sorts of questions that the technique could inform (Agarwal et al. 2020; Puschmann et al. 2020; Yu et al. 2021). Similarly, authors were encouraged to conduct alternative analyses using topic modelling in the future, such as on new datasets and different research questions.

In addition to demonstrating the use of topic models, many authors also provided a methodological framework targeted to their field. These frameworks tended only to offer a simple approach to topic modelling using a content analysis of topic word sets, considering specific disciplinary concerns such as using domain-specific dictionaries or enrolling subject matter experts for topic interpretation. Other researchers sought to integrate topic modelling into pre-existing methodologies. Integration was achieved in some instances using a mixed-methods approach (Jeong et al. 2019). Others augmented methods to accommodate and leverage topic modelling. Murashka et al. (2020) used topic modelling as one of three sampling strategies in a grounded theory approach. Brown (2019) introduced topic modelling for an auto-ethnographical analysis of self-generated SMD.

The lack of informed and structured methodological frameworks and the propensity for disciplines to insert topic modelling into pre-existing methodologies is problematic. For example, most studies adopt an exploratory or descriptive approach to topic modelling, asking high-level questions such as “What are the most common topics that are discussed and shared among Twitter users regarding online retail brands?” (Ibrahim and Wang 2019a). These articles are not less sophisticated than others. However, the insights gained are the result of a combination of techniques in addition to, rather than directly from topic modelling (Xu and Zhou 2020). Similarly, there is a lack of consistency across studies despite their similar approaches, specifically in how the number of topics is selected, how topics are evaluated, data preprocessing protocols and topic interpretation.

This area of research is still nascent, and there are promising examples of effective integration of topic modelling into well-known and rigorous methodologies. Le et al. (2019) stated in their investigations of the perceptions of cervical cancer to prevention strategies of Twitter users that their analysis of data was informed by grounded theory (Charmaz 2015) and that they followed Creswell’s mixed-methods approach (Creswell et al. 2011). They take care to adopt multiple strategies for theoretical sampling so as not to impose restrictions on the data they are exposed to. Their content analysis using topic modelling is one of these strategies. However, mixed-methods approaches are not an adequate or explanatory description of qualitative methodologies, including computational methods. Indeed, mixed methods appear to be used as a catchall for research methods otherwise unspecified. While this is not a new trend, it is one that was observed in this analysis and signals an opportunity to further develop systematic and reliable approaches to coding topics or to apply a qualitative methodology (Aslett et al. 2020; Hwang et al. 2020; Reyes-Menendez et al. 2020).

Still keeping with the concept of methodological rigour, the lack of validity and reliability of the topic modelling process was a concern to several authors who stated that there is an opportunity to develop further protocols to improve the legitimacy of this technique for SMD analysis (Puschmann et al. 2020). Some considered this the responsibility of topic modelling developers. For instance, Al-Ramahi et al. (2017) argues that the design of robust evaluation methods that instil trust in the topics is still an open challenge. Many authors were seen to adopt evaluation methods that are inappropriate for evaluation of topic modelling results for exploratory analysis, namely Perplexity (Chang et al. 2009; Lau et al. 2014). Others such as Aslett et al. (2020) view computational methods as providing ‘near-perfect reliability’ when human input is incorporated into the research design. The authors demonstrate that training annotators to verify topic quality by assessing the topic document-collection capitalises on the benefits of topic modelling for exploratory analysis. Comparison with other studies of the same phenomenon was suggested as a way to promote the external validity of the topics identified (Feldhege et al. 2020), though internal validity remained a concern (Kar 2020). Other studies addressed concerns around validity by assuring the reliability of their topic interpretations. Topic reliability was bolstered by employing multiple coders and calculating the inter-rater reliability (Cai et al. 2020; Jamison et al. 2020; Kirilenko et al. 2021) of the topic labels given to topics (topic word-sets or topic document-collections) by two or more annotators. However, the use of reliability measures does not address issues of topic quality as the reliability regards the coding schema and not topic construction and composition.

Interpreting only the topic words was the most common way topics were analysed. Here, the top ten terms ranked by probability are read, and the topic is given a label by one or more annotators (Liang et al. 2019; Kurten and Beullens 2021). In most studies, these topics were then described by drawing on the authors knowledge of the data set, the subject matter, or other contextual knowledge (Ibrahim and Wang 2019b). In some instances, labelled topics were grouped further and described as ‘themes’ (Pavlova and Berkers 2020). This method of interpretation is prominent in studies that employ topic modelling for content analysis, Infodemiology, or another type of exploratory analysis. For example, Abd-Alrazaq et al. (2020) in their study of tweets about the COVID-19 pandemic, asks, “What are the main topics posted by Twitter users related to the covid pandemic?”. For topic model developers, this is the assumed way that topic models are used, and indeed was the dominant method of interpretation and was conducted in 63.64% of studies. However, several studies raised concerns about possible biases that can arise with this approach (Brown 2019; Bérubé et al. 2020), as well as the depth of insights that are gained from it (Feldhege et al. 2020). Ibrahim and Wang (2019b) state that future research should address concerns around subjectivity in inferring meaning from topics.

Similarly, Hemmatian et al. (2019) and Hu et al. (2019) advise that the capacity for topic models to produce interpretable themes is merely the correlation of interpretability with the statistical features of the bag-of-words (BoW) representation of the documents. Some others rejected the capacity of topic models, specifically LDA, to generate any thematic understanding of texts, stating they were useful only to understand the most important words (Okon et al. 2020; Jamison et al. 2020). Hemmatian et al. (2019) warns that those using topic models should proceed with care when it comes to the epistemological assertions of topic models as important features of languages such as syntax and, thus, context, are lost in BoW representations.

Interestingly, those studies which analysed document-collections rather than word sets emphasised that rigorous thematic interpretation necessary to draw conclusions from topics and that it was not enough to simply label a topic word set or document set as a theme (Puschmann et al. 2020). Nizzoli et al. (2020) state that manual coding of topics can not only improve the accuracy, but the data that is generated can be used to refine unsupervised models further and enable more challenging predictive tasks in the future.

Topic model research recommendations: Approaches

(i) Topic modelling developers should have familiarity with how topics are interpreted and the epistemologies and methodologies that guide interpretation. This will inform design of topic models, performance measures, and validation of new performance measures.
(ii) Topic model research should, in many cases, not aim to target a breadth of settings but instead a well-defined set of applications, datasets and known interpretation protocols.
(iii) Application-driven design and/or a demonstrative case study should be adopted. This includes the specification of a use case.

### User knowledge

Aspects of user knowledge that are lacking, present an opportunity for topic model developers to bridge this knowledge gap as part of their research design. A prominent theme throughout many of the studies was that researchers, aiming to adapt discipline-specific methodologies to incorporate topic modelling, were reliant on alternative, often manual methods, to achieve a result that could have been achieved through the use of an already available computational tool.

A significant finding of this study was that LDA was used in the majority of studies (79.79%) even though it has been well documented in the empirical literature that LDA is sub-optimal for short texts such as social media (Hong and Davison 2010; Mehrotra et al. 2013; Cheng et al. 2014; Jónsso 2016), and there are many topic models that have been developed specifically for short and noisy social media texts (Qiang et al. 2020; Nugroho et al. 2020).

The choice of topic model should be informed by the features of the data, the size of the collection, the length of the documents, what the topics will be used for, and any other unique characteristics of the data such as noisiness or multiple languages. However, a review of the rationale provided for the reason LDA was adopted revealed that the primary reason was that it was seen to be used in other studies on the same research topic (Jamison et al. 2020; Agarwal et al. 2020), using the same type of data (Meyer et al. 2019; Hemsley et al. 2020), simply that LDA is the most popular topic model (Ibrahim and Wang 2019b; Nolasco and Oliveira 2020; Gurajala et al. 2019)

Aside from the known issues that LDA has with modelling sparse text, it was surprising to find that authors had chosen LDA given there are more appropriate models for their specific task such as temporal topic modelling (Dyda et al. 2019), hierarchical topic modelling (Liu 2020; Hwang et al. 2020), and in particular, multilingual topic models. Indeed, the primary limitation identified by authors was that they could not model a multilingual set of documents (Pavlova and Berkers 2020; Kar 2020).

While authors recognised that future work should incorporate more sophisticated methods, including temporal topic models (Dyda et al. 2019) and hierarchical topic models (Hemmatian et al. 2019), others mentioned the potential benefits of using deep learning methods and identified the use of neural topic models as an opportunity for future research (Gurajala et al. 2019; Bahja and Safdar 2020; Svartzman et al. 2020).

There is a knowledge gap in tuning and optimising topic models for use in an applied context. Despite the significant impact that hyperparameter settings have on the topics produced, few studies addressed this task beyond setting the number of topics (Brown 2019; Chan et al. 2020). The sensitivity of topic modelling to *K* was not well understood and was identified as a limitation of using topic models by several authors (Al-Ramahi et al. 2017; Gurajala et al. 2019). Others acknowledge the implications of *K* on topic interpretation but argued that it was challenging to optimise the number of topics which many highlighted as a limitation (Lock and Pettit 2020).

A troubling trend in the selection of *K* was identified. In some instances, authors selected *K* by using the same value for *K* as previous studies and did not conduct any assessment of different values for *K* (Zhu et al. 2020; Nizzoli et al. 2020; Puschmann et al. 2020). This method is sub-optimal and risks the formation of quality topics as it does not account for the differences in dataset composition and size. Indeed, there were instances where *K* was chosen based on it being trialled on a different data set, in some cases from a different social media platform (Nizzoli et al. 2020; Abd-Alrazaq et al. 2020; Zhu et al. 2020). Others referred to the empirical literature, selecting the same hyperparameter values as those reported in the empirical literature (Joo et al. 2020; Yan et al. 2020; Zhang et al. 2021), which are not typically optimised in development studies presenting a new topic model. Few studies reported the alpha and beta hyperparameters for LDA, for example, and even fewer engaged in tuning these (Brown 2019; Chan et al. 2020) or the number of iterations and chunk size (Ibrahim and Wang 2019b; Zhai et al. 2020).

Evaluation of models was rarely completed as a distinct step from the selection of *K*. While the interpretation of models was conducted separately, no other steps were taken to repeat any modelling to optimise topic quality beyond selecting from a set of topics modelled under different values of *K*. Of those studies that did employ a form of evaluation, the most common form was through the use of inter-rater reliability measures such as Krippendorff’s $\alpha$ (Reyes-Menendez et al. 2020; Peres et al. 2020) and Cohen’s $\kappa$ (Zhou and Na 2019; Kwon et al. 2019).

One important finding concerned the inconsistency between evaluation measures used to select *K* (Brown 2019). As discussed previously, different articles cited different evaluation measures. However, the R package ‘ldatuning’ (Murzintcev 2020) was used by a relatively large number of studies to optimise (Hu et al. 2019; Gregoriades and Pampaka 2020; Zhai et al. 2020; Zhang et al. 2020; Xu and Xiong 2020). The ‘ldatuning’ package offers four methods to estimate the optimal number of topics: maximising divergence values produced from symmetric KL-Divergence of salient distributions derived from these matrix factors (Arun et al. 2010); minimising distance among topics and their densities (Cao et al. 2009); Jensen-Shannon divergence for topic similarity (Deveaud et al. 2014); and minimising perplexity, the log-likelihood of unseen words. Perplexity is a common evaluation measure in topic model evaluation. It is used to infer the effect of changes to the number of topics and to determine how well a probability distribution (model) predicts a sample (Griffiths and Steyvers 2004). It was not clear which of these measures authors favoured, and some reported being challenged by the lack of convergence between them (Zhai et al. 2020; Zhang et al. 2020).

A concern here is that these measures are not intended to provide a basis to optimise *K* in these settings. Moreover, common measures such as perplexity are well known to be inadequate measures of topic quality in terms of interpretability (Chang et al. 2009; Lau et al. 2014). Coherence scores have been shown to be poor estimates for the quality of topics generated from tweets using LDA (Doogan and Buntine 2021). Performance measures typically seen in the evaluation of novel algorithms were employed in studies to determine the optimal number of topics. Many authors combined these measures with a manual inspection of the topics. However, this process is compromised by the narrow range of topics chosen to be trialled, the lack of direction in how many topics should be expected for the size of the document collection, and the expectation that some authors had of the topic representation. For example, the number of topics modelled was inconsistent across the collection relative to the number of modelled documents. Very small numbers of topics were modelled for relatively large document collections (Wicke and Bolognesi 2020; Hemsley et al. 2020). Generally, however, the value of *K* was within the bounds of what was acceptable for the number of documents, and a small number of studies did acknowledge this factor (Kirilenko et al. 2021).

Several studies promoted triangulation of evaluation measures to produce interpretable, meaningful and intuitive topics (Reyes-Menendez et al. 2020; Doogan et al. 2020). In determining the optimal *K*, Fischer-Preßler et al. (2019) considered the size of the documents, document collection, and object of study, which in this research, were tweets about a specific event collected via hashtag filtering. They recognised that larger models would not be appropriate for a smaller collection of 50,000 tweets. They then evaluated $K = 10-40$ and isolated $K = 10$ and 20 as candidates based on calculated coherence and exclusivity. The top 50 terms in each topic and top 50 documents were examined and labelled. The choice of 20-topics was made based on their experience, with the authors stating that these topics were more intuitive than the others. In this way, qualitative methods are supported by quantitative guidance.

Preprocessing was inconsistent between articles to a greater degree than expected. A rationale for their choices was under-reported, signalling an under-appreciation or lack of understanding of the importance of data treatment for the topic formation and semantic meaning (Xue et al. 2020b, 2020c). When authors did provide some basis for these choices, we found that they were mostly informed by the empirical literature, which was not on topic modelling (Ha et al. 2017; Kirilenko et al. 2021), or not relevant to the application context (Chae 2019; Dyda et al. 2019; Berg et al. 2020; Zhai et al. 2020; Valdez et al. 2020; Yu et al. 2021). Others referred to research using topic modelling previously conducted in their field (Hacker et al. 2020). Finally, those authors who have previously used topic models were seen to adopt the same data treatment methods (Ibrahim and Wang 2019b).

Of significant concern was the potential to corrupt downstream or secondary analysis conducted using the data. In some articles, data was not re-processed to cater to sentiment analysis which has different requirements to topic models (Zhai et al. 2020; Xue et al. 2020a). Indeed, some studies incorrectly reported the rationale for preprocessing as being a way to reduce bias in topic interpretation (Pavlova and Berkers 2020). Another mistaken assumption was regarding the removal of punctuation and special characters.

The choice to stem or lemmatise tokens illustrates the lack of understanding of the relationship between model behaviour, data processing, and human interpretation. English words, particularly verbs, have multiple forms which are context-dependent. Stemming is the process by which the term is reduced to its *stem* word. For example, the stem of ‘started’ and ‘starting’ is ‘start’. While the tense has changed, the meaning of the term remains. However, there are instances in which the application of stemming will alter the meaning of the word, such as the adjective ‘boring’ where the stemmed word is the verb ‘bore’, which has a very different meaning to ‘boring’. Here, stemming has introduced a lexical ambiguity as the term has multiple meanings. This makes topics harder to interpret and will result in less specific topics in the first place. Additionally, stemming creates terms that have no meaning, such as in the case of ‘stay’, which is stemmed to ‘stai’. The presence of these non-sensical words will hinder human interpretation of topics.

Although stemming (Jin et al. 2021), and occasionally both stemming and lemmatisation, are still commonly adopted in the topic development literature (Erfanian et al. 2022), stemming should not be used for topic modelling as model inference will assign any words that have the same stem to the same topic. This morphological conflation may result in an improved joint probability of documents but will not improve the quality of the model and may even damage it (Schofield and Mimno 2016; Schofield et al. 2017). Stemming has been shown to affect the accuracy of held-out predictive likelihood-based evaluations of models (Schofield et al. 2017). Not only does stemming hinder interpretation, but it also produces topics based on documents that do not share a true semantic relationship. In addition, articles using LDA for modelling for short, noisy texts already compromise topic quality, have been shown to adopt stemming and perplexity as a singular evaluation metric to infer the quality of topics (Qi et al. 2020) or to determine the optimal number of topics *K* (Kirilenko et al. 2021). We note that the authors of applied papers identified several limitations of topic modelling that could be resolved by improving data handling and topic model selection.

Topic model research recommendations: User knowledge

(i) Experimental studies must be conducted to bridge the gap between theoretical and applied work, as applied researchers may not understand the model behaviours responsible for the model output.
(ii) Increased transparency of experimental settings, parameters, statistical presentation of performance, preprocessing and the limitations of novel topic models is required.
(iii) Undertake interdisciplinary collaboration to benefit the construction and development of domain-specific methodological frameworks for applied researchers.
(iv) Efforts should be made to investigate and explicitly articulate the limitations of a topic model within the context of an applied setting.
(v) Developers should regularly engage with the applied literature to learn the needs of researchers using topic models and what is not working.

### Advancing research

This systematic review aimed to provide insight into the applications of topic models. This information is useful to topic model developers to further understand the needs of those using topic modelling for their research, identify where research has underperformed when applied to real-world settings, and possible research gaps that require further attention. The section provides an overview of the areas for possible research advancement by both those who develop topic models and those who use them.

The reviewed papers consistently stated that the validity of topic modelling as a research approach was challenging to promote (Hemmatian et al. 2019; Feldhege et al. 2020; Bérubé et al. 2020; Kar 2020). The validity of topics is important as it instils trust in the research outcomes to the broader community. Given that the use of these outcomes can inform critical work such as the diagnosis of mental health illnesses (Li et al. 2020b), support (Kwon et al. 2019), or public health responses (Yu et al. 2021). Should the insights provided by such studies be misinformed or inaccurate, there is a risk that actions informed by these studies could adversely affect real-world outcomes. We identified that validity promotion was challenging because the evaluation measures used did not necessarily correlate to contextually meaningful topics.

A significant finding of this study was the variety of evaluation measures employed, particularly when selecting *K*. Our analysis revealed that held-out likelihood (Perplexity) (Griffiths and Steyvers 2004) was the most common measure used to evaluate the models (22.04%) (Kirilenko et al. 2021; Zhang et al. 2021). This was an interesting observation given that it is well documented in the topic modelling literature that perplexity is not an accurate measure of semantic interpretability (Chang et al. 2009) and that perplexity should not be used as a singular measure of topic quality (Lau et al. 2014).

The authors of the reviewed papers have highlighted that the lack of direction on the use of evaluation measures to demonstrate the validity of their findings as a limitation. Authors adopted alternative strategies to demonstrate validity such as comparison findings to prior studies to promote external validity, and by calculating inter-rater reliability to demonstrate the reliability in topic interpretations (Hemmatian et al. 2019; Feldhege et al. 2020; Bérubé et al. 2020; Kar 2020) have used the However, these tools do more to promote trust in the interpretations of the topic by the researchers than in the quality of the topics being interpreted. Feldhege et al. (2020) reported that topic modelling, in this case, LDA, was chosen for their investigation into Reddit forums on depression as it promised high levels of semantic coherence, which they understood to be correlated to topic interpretability and agreement with human evaluations. However, they found that topics were still ambiguous as they lacked the context provided by the tone and style of the posts. Others report that these measures inform the construction of topics that highlight important words but do not provide a thematic understanding of the texts (Okon et al. 2020). While authors have used their own disciplinary tools to promote the validity of their qualitative outcomes, there is not yet a consensus on how the validity of the topics proposed to represent the underlying document collection can be achieved.

Indeed, the rationale of evaluation, to demonstrate the performance of a topic model, was conflated in almost all papers as a means to select an optimal number of topics to model. This dual-use is problematic, but it does reveal that researchers that use topic models require new quantitative ways to instil trust in the topics tailored to the use case for which they are employed. For example, classification accuracy, which has been queried in some articles (Xin and MacEachren 2020; Nizzoli et al. 2020), is not an adequate measure of the performance of models to be used as exploratory devices. As such, evaluation measures used in topic model development, specifically coherence^9^, perplexity, purity, and classification accuracy, may not inform the depth of meaning and usability.

It is currently difficult to assess the outcomes of topic modelling as an unsupervised technique for exploratory analysis used to uncover patterns in textual data. It is still an open question whether effective evaluation procedures can be designed so that the researchers can be confident of the themes identified in texts that they have never seen (Al-Ramahi et al. 2017). However, future research could leverage the findings of this study with regards to what a quality topic looks like according to researchers that employ topic modelling in their studies. In addition to interpretability, exclusivity and stability were seen as qualities of topics that authors looked for when selecting *K*.

Finally, we identified that researchers who make use of topic modelling are highly reliant on software packages that are easy to use and well known. We hypothesise that this may be a primary reason that LDA has been used in the majority of studies, as these well-established tools all use LDA as their default topic model. Given that authors expressed a desire to implement more sophisticated modes, specifically neural topic models, accessible and user-friendly tools are needed to support the broader research community in using these techniques.

Preprocessing is being conveyed as a one-size-fits-all in the empirical literature and what is reported is different across studies. Part of this is because actual qualitative interpretation is not conducted in empirical studies, and so little attention is paid to the actual interpretability of topics, or rather the ability for them to convey the meaning which is truly representative of that held by the sample of documents. Another reason is the lack of detail provided in empirical documentation. Preprocessing requires further attention and documentation. This may also improve the trust in topic models, making it easier to determine if a topic model reports improved performance as a function of the algorithm or the data treatment. For example, overly aggressive reduction of the vocabulary through stemming is known to improve performance as the probability space of the model is reduced, thus producing increased performance scores (Schofield et al. 2017). If the trade-off between meaningful topics and vocabulary reduction is not acknowledged, it may be that topic models scored this way may underperform when stemming or lemmatisation is conducted in a way that preserves the interpretability of a topic necessary for applied studies.

Topic model research recommendations: Research advancement

(i) The validity of topic modelling should be addressed. The focus should be given to the development and validation of alternative performance measures which reflect the needs of researchers who are applying topic models to SMD.
(ii) Alternative measures of performance that are in line with the needs and preferences of researchers applying topic models would be better suited as benchmark measures for the evaluation of new topic models.
(iii) User-friendly implementation (tools and software) is required to ensure uptake of new models and approaches. Efforts should be made to make code more accessible. One example would be appropriate algorithms or at least methodological support to ‘select’ the number of topics *K*.
(iv) Further investigation of the impacts of data features, preprocessing, and data quality on model performance is needed.

## Conclusion

This SLR of existing literature on topic modelling applications for social media analysis, focused on how the topic modelling field can build on the literature from other disciplines. It defines several directions and recommendations for short text topic modelling research, particularly those geared towards social media investigations.

To ensure effective uptake and application of topic modelling research in the future, we conclude that the field must participate and drive in the translation of its output to applied research. This could begin by developing a refined understanding of applied topic modelling intentions and broadening the empirical focus of the field’s research and their familiarity with the types of theoretical and epistemological frames through which topic models are interpreted. It should also expand its analytic capacity to address discipline-specific needs. Furthermore, there is ample room for topic modelling research to explicitly connect topic model development to contemporary applications of topic modelling structured around the various research paradigms by which applied work is conducted.

The exponential increase in research that employs computational methods is significant. Medical informatics, public health, communications, information systems, and information sciences are among the fields where topic modelling research is highly valued. It is worth noting that topic modelling has the potential to drive clinically oriented research and, as a result, patient outcomes in the medical fields. This systematic review also discusses the implications for applied research. To be more specific, the sub-optimal practices should be addressed to bolster the validity and impact of applied topic modelling research. The clarification of these may aid practitioners in improving their research design, ultimately elevating the trustworthiness of computational methods. In this sense, our study directs topic modelling researchers to consider the critical capabilities required for impactful application of topic models and calls the attention of practitioners to those aspects of practice that may impede the success of topic modelling for social media analysis.

One limitation of this study is that it may not provide a comprehensive picture of topic modelling applications. Given the recent explosion of peer-reviewed articles, the research design required inclusion criteria, which reduced the volume of potentially relevant literature reviewed. Despite this limitation, we believe that this SLR provides the promised visibility over applied topic modelling research practises for social media data in the cross-disciplinary literature. We hope our work inspires more systematic efforts to conduct application-driven research on topic modelling development.

## Supplementary Information

Below is the link to the electronic supplementary material.

Supplementary file 1 (pdf 846 KB)

## Data Availability

The datasets created and analysed for this article are included within the article and its supplementary information files.

## Footnotes

1 This paper uses the term ‘researchers’ to describe those who use topic models for social media analysis. The term ‘developers’ describes researchers who develop novel topic models.

2 SCImago Journal Rank https://www.scimagojr.com/.

3 Source Normalized Impact per Paper.

4 See http://portal.core.edu.au/jnl-ranks/.

5 A management platform for systematic review https://www.covidence.org/.

6 Research Areas were adapted from WoS: Life Sciences & Biomedicine, Social Sciences, Physical Sciences, Arts & Humanities Technology and Multidisciplinary Science.

7 WoS research areas https://images.webofknowledge.com/images/help/WOS/hp_research_areas_easca.html Multidisciplinary Sciences was added to this schema to capture those publications with this WoS disciplinary category.

8 This was informed by inspection of topics, an inspection of source code, or through a declaration by the authors.

9 The coherence measures identified were *C*_*Umass*_, *C*_*PMI*_, *C*_*V*_, *C*_*UCI*_, *C*_*NPMI*_.

## Code availability

Code availability is not applicable to this work.
