# Supplementary material for: A systematic review of the use of topic models for short text social media analysis
Source: Artif Intell Rev. 2023 May 1:1–33. Online ahead of print. doi: 10.1007/s10462-023-10471-x (PMC10150353; doi:10.1007/s10462-023-10471-x)
Supplement: Supplementary file 1 — (pdf 846 KB) [file 10462_2023_10471_MOESM1_ESM.pdf]

Appendix 1

Appendix 1.2 Search Strings and Keywords

Search strings included a combination of the terms: a) "TOPIC MODEL*", or a specific topic model; and b) "SOCIAL MEDIA", "MICROBLOG*", "TUMBLELOG*", "USER GENERATED CONTENT", "UGC", or a specific social media site, and/or c) either "SHORT-TEXT*", "SHORT TEXT*", or "SPARS*".

An example of a search string for an EBSCO database search query"

((TS=("Wechat" OR "weibo" OR "Jaiku" OR "Plurk" OR "Tumblr" OR "wooxie" OR "LinkedIN" OR "Pintrest" OR "Mastodon" OR "Reddit" OR "Facebook" OR "Gab" or "Instagram" OR "GNU Social" OR "renren" OR " xiaonei" OR "Flickr" OR "diaspora" OR "twister" OR "Pleroma" OR "identi.ca" OR "micro.blog" OR "Yammer" OR "tout" OR "Misskey")AND TS=("Topic model*")))

Specific social media platforms queried were:

1. Academia.edu
2. BitClout
3. Classmates
4. Diaspora
5. Discord
6. Facebook
7. Flickr
8. Friendster
9. Gab
10. GNU Social
11. identi.ca
12. Instagram
13. Jaiku
14. LinkedIn
15. Mastodon
16. micro.blog
17. Misskey
18. Pleroma
19. Pintrest
20. Plurk
21. Qzone
22. Reddit
23. RenRen (Xiaonei pre Aug 2009)
24. Sina Weibo
25. Soup.io
26. TikTok
27. Tout
28. Tumblr
29. Twister

30. Wooxie
31. Wykop.pl
32. Yammer
33. WeChat
34. Xing

Explanatory notes for Figure 1: Using WoS, the search string TI = (topic model*) was used to query results. These were restricted to document types: Article, Meeting (conference papers) and early access. The results were further restricted to those published in Computer Science journals. There were 2,604 articles returned. A citation analysis was conducted. The sum of times these articles were cited was 26,741 from 17,529 citing articles, and 22,501 without self-citation from 16,104 articles. The average citation per item was 10.27 citations.

Appendix 2

Appendix 2.1 Data Extraction Template

DATA EXTRACTION

General information

BibTex Ref ID

Where is the institution(s) of the authors located?

If there are multiple locations, choose 'other' and list these.

- ○ Australia
- ○ Canada
- ○ China
- ○ India
- ○ Ireland
- ○ Qatar
- ○ United Kingdom
- ○ United States
- ○ Singapore
- ○ Saudi Arabia
- ○ Germany
- ○ South Korea
- ○ The Netherlands
- ○ Sweden
- ○ Spain
- ○ Other

Clear above selection

What disciplines do the researchers come from?

Look at the faculties or departments listed and summarise this. Detail is helpful, but if there are too many, please group them, e.g., Informatics, network systems, and Software engineering can be grouped under Computer science.

Characteristics of included studies

Aims and Objectives

What were the aims/objectives of the study?

Be specific, the key point is to identify the 'why' behind the actions. What knowledge is this going to contribute?

What were the research questions/Hypotheses ?

If there was none then write 'None'

Methodology

What was the main methodology (if mentioned)?

For example, Sentiment analysis and topic modelling.

What cohort/population was being studied?

For example, posts by users of a cancer support forum on Reddit who self-identified as patients with non-Hodgkin and Hodgkin Lymphoma. These posts were between 2014-2017 and contained the term 'Carmustine'

What rational (if any) was given for this cohort/population being chosen?

Explain the reasoning behind the choice. For example, capturing posts between 2014-2017 encompassed a time after Carmustine was available to Hodgkin lymphoma and non-Hodgkin lymphoma patients. Key terms 'Hodgkin', 'non-Hodgkin' and 'lymphoma' were used to filter out posts by patients with other cancers that the drug is used to treat.

Which of the following is the best match for the study design?

If the study is not adequately described by any of these or fits more than one of these categories, choose 'other' and describe this.

- ○ Domain specific methodology
- ○ Application Framework - general
- ○ Application Framework - domain specific task
- ○ Qualitative research - case study
- ○ Qualitative research - exploratory
- ○ Comparative evaluation of techniques
- ○ Comparative evaluation of cohort/population
- ○ Survey or review
- ○ Other

Clear above selection

Data

What social media data was modelled?

If there are more than one, choose the other option and add these as free text.

- ○ Twitter
- ○ Reddit
- ○ Facebook
- ○ Instagram
- ○ Weibo
- ○ Telegram
- ○ Blued
- ○ Flickr
- ○ Instagram
- ○ YouTube (Comments)
- ○ Other

Clear above selection

What rationale was given for this data being chosen?

What was the number of documents modelled?

Some studies model documents in batches. Please describe this if this is true of the study being reviewed. NOTE: This is not the number collected, but the number modelled after pre-processing and batching (if done(.

Lowest number of documents modelled

If the number of documents is above 500,000 please enter the number.

- ○ 0 - 1,000
- ○ 1,001 - 10,000
- ○ 10,001 - 20,000
- ○ 20,001 - 40,000
- ○ 40,001 - 60,000
- ○ 60,001 - 80,000
- ○ 80,001 - 100,000
- ○ 100,001 - 150,000
- ○ 150,001 - 200,000
- ○ 200,001 - 250,000
- ○ 250,001 - 300,000
- ○ 300,001 - 400,000
- ○ 400,001 - 500,000
- ○ 500,000 +
- ○ Other

Clear above selection

Was it mentioned if documents standardised to lower-case?

- ○ Yes
- ○ No
- ○ NA
- ○ Other

Clear above selection

Was it mentioned if punctuation was removed?

- ○ Yes
- ○ No
- ○ NA
- ○ Other

Clear above selection

Was it mentioned if retweets were removed?

- ○ Yes
- ○ No
- ○ NA
- ○ Other

Clear above selection

Was it mentioned if slang/abbreviations was standardised?

- ○ Yes
- ○ No
- ○ NA
- ○ Other

Clear above selection

Was it mentioned if duplicates were removed?

- ○ Yes
- ○ No
- ○ NA
- ○ Other

Clear above selection

Was it mentioned if special characters were removed?

- ○ Yes
- ○ No
- ○ NA
- ○ Other

Clear above selection

Was it mentioned if emojis/emoticons were removed?

- ○ Yes
- ○ No
- ○ NA
- ○ Other

Clear above selection

Was it mentioned if numbers were removed?

- ○ Yes
- ○ No
- ○ NA
- ○ Other

Clear above selection

Was it mentioned if the key words used to retrieve posts were removed from the documents?

- ○ Yes
- ○ No
- ○ NA
- ○ Other

Clear above selection

Was it mentioned if non-english (or other language of focus) documents were removed or filtered?

- ○ Yes
- ○ No
- ○ NA
- ○ Other

Clear above selection

Was it mentioned if any of the following were conducted:

- ○ Lemmitisation
- ○ Stemming
- ○ Both
- ○ NA
- ○ Neither
- ○ Other

Clear above selection

Was it mentioned if stopwords were removed?

- ○ Yes
- ○ No
- ○ NA
- ○ Other

Clear above selection

Was it mentioned if bi-grams were generated?

- ○ Yes
- ○ No
- ○ NA
- ○ Other

Clear above selection

Was it mentioned if tri-grams were generated?

- ○ Yes
- ○ No
- ○ NA
- ○ Other

Clear above selection

Was it mentioned if documents were tokenised?

- ○ Yes
- ○ No
- ○ NA
- ○ Other

Clear above selection

Was it mentioned if PoS-tagging was conducted?

- ○ Yes
- ○ No
- ○ NA
- ○ Other

Clear above selection

Was it mentioned if documents were removed if they were a certain length?

○ Yes
○ No
○ Other

Clear above selection

If either low of high frequency terms were removed, or documents of a certain length were removed, explain the method. If not applicable type 'NA'.

Was there an explanation of the reasons for or justification of the pre-processing?

Were any tools used for pre-processing?

Were any specific tools mentioned that were used in the preparation of the data e.g. NLTK, Spacy?

Comments on pre-processing

Specifically, if you know that they conducted stemming after viewing the example topics (if given) but they did not mention stemming, write that here.

Topic modeling

Why was topic modelling adopted?

What reasons did the authors give?

Were any specific topic modelling tools used?

○ 'sklearn' in Python
○ Gensim
○ MALLET
○ TopicViz
○ 'TopicModels' in R
○ Other

Clear above selection

What topic modelling algorithm was used?

○ LDA
○ BTM
○ MetaLDA
○ CorEx
○ MG-LDA
○ NMF
○ TweetLDA
○ STM
○ Author-LDA
○ Other

Clear above selection

Why did the authors choose this specific algorithm?

Why did the authors choose this specific algorithm?

Were any parameters mentioned? Please give details.

Any alpha and beta values, k, number of iterations etc.

What number of topics was k set at?

How was the number of topics chosen?

Did they use coherence scores, which ones? Did they eye-ball the topics or tweets? Did they use software such as LDA tuning in R?

What was done with these topics? And/or How were topics interpreted?

For example, did the authors simply read the top 10 terms in the topic an interpret these? Did they read the underlying collection? or did they conduct some other form of analysis?

Were any other techniques used on the data?

- ○ Sentiment analysis
- ○ Social Network Analysis
- ○ Author classification tasks
- ○ Other

Clear above selection

Details of other techniques

Specifically the order, but also if anything done in topic modelling influenced the outcome of these other techniques e.g. they conducted sentiment analysis on the tweets from each topic. They re-processed the data (or more likely did not).

Evaluation

How was the quality of the topic models evaluated?

Both qualitative measures such as IRR and quantitative measures such as NPMI are examples. Discussion between SME is another. This is different from the method used to choose K.

Other considerations

Was ethics, privacy or consent mentioned?

- ○ Yes, with notice of ethics approval
- ○ Yes (ethics), but there was no notice of ethics approval
- ○ Yes (privacy)
- ○ Yes (consent)
- ○ No to all
- ○ Other

Clear above selection

What limitations (related to topic modelling) were mentioned?

They must specifically mention topic modelling and a limitation of using it, or the specific algorithm they used.

What opportunities or future directions (relating to topic modelling) were mentioned?

Are topics available?

- ○ Yes
- ○ No
- ○ Other

Clear above selection

Any specific comments or points of interest about the paper should be entered here. Otherwise write 'None'.

Appendix 2.2 Disciplinary Classification Process

The articles were labelled according to the 1-4 WoS discipline categories provided by WoS for the journal published in, which were cross-checked against those assigned to each publication in Scopus®[10]. These disciplinary categories informed a single category prescribed to each journal. When two categories appeared equally appropriate, the category was chosen based on a review of the recent articles from that journal.

Life Sciences & Biomedicine condensed disciplinary categories

Public Health

- Public Health, Environmental & Occupational Health

Medical Informatics

- Medical & Computational Biology
- Medical Informatics

Medicine & Health Care Sciences

- Medical & Computational Biology
- Biotechnology & Applied Microbiology
- Dermatology
- Infectious Diseases
- Medical, Legal
- Medicine, General & Internal
- Medicine, Geriatrics
- Medicine, Research & Experimental
- Nursing
- Pediatrics
- Psychiatry
- Substance Abuse

Physical Sciences condensed disciplinary categories

Environmental Science

- Environmental Science
- Environmental Studies

Social Sciences condensed disciplinary categories

Business & Management

- Business

[10]Eight articles did not appear in the WoS collection and were categorised according to the Scopus® disciplinary labels

- Management
- Operations Research & Management Science

Political Science & Policy

- Political Science
- Public Administration

Psychology

- Psychology, Applied
- Psychology, Experimental
- Psychology, Multidisciplinary
- Psychology, Social

Sociology & Social Science

- Cultural Studies
- Social Sciences, Biomedical
- Social Sciences, Interdisciplinary
- Social Sciences, Law
- Sociology

Transportation & Urban Studies

- Transportation
- Urban Studies

Technology condensed disciplinary categories

Computer Science

- Computer Science, Information Systems
- Computer Science, Interdisciplinary
- Computer Science, Theory & Methods

Engineering

- Engineering, Environmental
- Engineering, Industrial

Remote Sensing & Geosciences

- Geosciences, Multidisciplinary
- Remote Sensing

Appendix 3

Appendix 3.1 Journals of Surveyed Articles

Table 6 Journals of Surveyed Articles

Journal	Research Category	Count	Prop.
J. Med. Internet Res.	Medical informatics	29	15.34%
PLOS ONE	Multidisciplinary sciences	9	4.76%
Int. J. Disaster Risk Reduct.	Remote sensing & Geosciences	5	2.65%
Int. J. Inf. Manage.	Information science & Library science	5	2.65%
IEEE Access	Computer science	4	2.12%
Inf. Process. Manage.	Computer science	4	2.12%
Info. Commun. Soc.	Information systems	4	2.12%
Online Inf. Rev.	Information science & Library science	4	2.12%
Comput. Hum. Behav.	Psychology	3	1.59%
Digit. Journal.	Communication	3	1.59%
Telemat. Inform.	Information science & Library Science	3	1.59%
Am. J. Public health	Public, Environmental & Occupational health	2	1.06%
Comput. Environ. Urban Syst.	Environmental science	2	1.06%
First Monday	Social sciences other topics	2	1.06%
Health Commun.	Communication	2	1.06%
Int. J. Commun.	Communication	2	1.06%
Int. J. Environ. Res. Public Health	Public, Environmental & Occupational health	2	1.06%
Int. J. Geogr. Inf. Sci.	Geography	2	1.06%
Internet Res.	Business & Economics	2	1.06%
J. Affect. Disord.	Psychiatry	2	1.06%
J. Gen. Intern. Med.	Health care sciences & services	2	1.06%
J. Inf. Sci.	Information science & Library Science	2	1.06%
New Media Soc.	Communication	2	1.06%
Public Relat. Rev.	Business & Economics	2	1.06%
Soc. Med. Soc.	Communication	2	1.06%
Soc. Netw. Anal. Min.	Computer science	2	1.06%
Soc. Sci. Med.	Public Health & Public, Env. & Occup. health	2	1.06%
ACM Trans. Manag. Inf. Syst.	Computer science	1	0.53%
AIDS Behav.	Public, Environmental & Occupational health	1	0.53%
Am. Behav. Sci.	Psychology	1	0.53%
Am. Sociol. Rev.	Sociology	1	0.53%
Ann. Am. Assoc. Geogr.	Geography	1	0.53%
Ann. Oper. Res.	Operations research & Management science	1	0.53%
Appl. Geogr.	Geography	1	0.53%
Behav. Res. Methods	Psychology	1	0.53%
Cartogr. Geogr. Inf. Sci.	Geography	1	0.53%
Case Stud. Transp. Policy	Geography	1	0.53%
Chaos Solitons Fractals	Physics	1	0.53%
Child Abuse Negl.	Family studies	1	0.53%
Cities	Urban studies	1	0.53%
Comput. Biol. Med.	Mathematical & Computational biology	1	0.53%
Comput. Ind. Eng.	Computer science	1	0.53%
Comput. Secur.	Computer science	1	0.53%
Cult. Stud.-Crit. Methodologies	Cultural studies	1	0.53%
Cyberpsychol. Behav. Soc. Netw.	Psychology	1	0.53%
Decis. Support Syst.	Computer science	1	0.53%
Electron. Commer. Res. Appl.	Business & Economics	1	0.53%
Electron. Libr.	Information science & Library science	1	0.53%
Env. Plan. B-Urban Anal. City Sci.	Geography	1	0.53%
Eur. J. Commun.	Communication	1	0.53%
Eur. J. Inf. Syst.	Information systems	1	0.53%
F1000Research	Biochemistry & Molecular biology	1	0.53%
Forensic Sci. Int.	Criminology & Psychology	1	0.53%
Front. Psychiatry	Psychiatry	1	0.53%
Front. Psychol.	Psychology	1	0.53%
Futur. Gener. Comp. Syst.	Computer science	1	0.53%

Geo. Spat. Inf. Sci.	Remote Sensing & Geosciences	1	0.53%
Gov. Inf. Q.	Information Systems	1	0.53%
Health Inf. Sci. Syst.	Medical Informatics	1	0.53%
Heliyon	Business & Economics	1	0.53%
Human Vaccines Immunother.	Immunology	1	0.53%
Ind. Mark. Manag.	Business & Economics	1	0.53%
Inf. Manage.	Information Systems	1	0.53%
Inf. Syst. Front.	Information Systems	1	0.53%
Infect. Control. Hosp. Epidemiol.	Public, Environmental & Occupational Health	1	0.53%
Int. J. Advert.	Business & Economics	1	0.53%
Int. J. Digit. Earth	Remote Sensing & Geosciences	1	0.53%
Int. J. Eating Disord.	Psychiatry	1	0.53%
Int. J. Med. Inform.	Medical Informatics	1	0.53%
Int. J. Nurs. Pract.	Nursing	1	0.53%
Int. J. Press/Politics	Government & Law	1	0.53%
J. Addict. Dis.	Substance Abuse	1	0.53%
J. Addict. Med.	Substance Abuse	1	0.53%
J. Advert.	Business & Economics	1	0.53%
J. Am. Acad. Dermatol.	Dermatology	1	0.53%
J. Am. Med. Inf. Assoc.	Medical Informatics	1	0.53%
J. Appl. Psychol.	Psychology	1	0.53%
J. Biomed. Inform.	Medical Informatics	1	0.53%
J. Clean. Prod.	Construction & Building Technology	1	0.53%
J. Comput. Sci.	Computer Science	1	0.53%
J. Comput.-Mediat. Commun.	Cultural Studies	1	0.53%
J. Gerontol. B Psychol. Sci. Soc. Sci .	Psychology	1	0.53%
J. Health Commun.	Public, Environmental & Occupational Health	1	0.53%
J. Inf. Technol. Politics	Business & Economics	1	0.53%
J. Infect. Public Health	Public, Environmental & Occupational Health	1	0.53%
J. Int. Market.	Government & Law	1	0.5%
J. Intercult. Commun. Res.	Cultural Studies	1	0.53%
J. Manage. Eng.	Construction & Building Technology	1	0.53%
J. Med. Sys.	Medical Informatics	1	0.53%
J. Nurs. Scholarsh.	Nursing	1	0.53%
J. Prof. Nurs.	Nursing	1	0.53%
J. Serv. Mark.	Business & Economics	1	0.53%
Mar. Pollut. Bull.	Environmental Sciences & Ecology	1	0.53%
Media Commun.	Communication	1	0.53%
Nicotine Tob. Res.	Public, Environmental & Occupational Health	1	0.53%
Open Forum Infect. Dis.	Public, Environmental & Occupational Health	1	0.53%
Paediatr. Perinat. Epidemiol.	Obstetrics & Gynecology	1	0.53%
Pers. Individ. Differ.	Psychology	1	0.53%
PLOS Biol.	Biochemistry & Molecular biology	1	0.53%
Policy Stud. J.	Government & Law	1	0.53%
Prof. de la Inf.	Information Science & Library science	1	0.53%
Public Transp.	Transportation	1	0.53%
Sci. Total Environ.	Environmental Science	1	0.53%
Scientometrics	Information Science & Library science	1	0.53%
Soc. Indic. Res.	Government & Law	1	0.53%
Soc. Sci. Comput. Rev	Computer Science	1	0.53%
Subst. Abus.	Substance Abuse	1	0.53%
Telecommun. Policy	Communication	1	0.53%
Tour. Manag.	Business & Economics	1	0.53%
Travel Behav. Soc.	Transportation	1	0.53%
Vaccine	Immunology	1	0.53%

Appendix 4

References

[1] Mohammad A Al-Ramahi, Jun Liu, and Omar F El-Gayar. Discovering design principles for health behavioral change support systems: A text mining approach. *ACM Transactions on Management Information Systems (TMIS)*, 8(2–3):1–24, 2017.

[2] Alicia L Nobles, Eric C Leas, Carl A Latkin, Mark Dredze, Steffanie A Strathdee, and John W Ayers. #HIV: Alignment of HIV-related visual content on Instagram with public health priorities in the US. *AIDS and Behavior*, 24:2045–2053, 2020.

[3] Saif Shahin and Zehui Dai. Understanding public engagement with global aid agencies on Twitter: A technosocial framework. *American Behavioral Scientist*, 63(12):1684–1707, 2019.

[4] Tim K Mackey, Janani Kalyanam, Takeo Katsuki, and Gert Lanckriet. Twitter-based detection of illegal online sale of prescription opioid. *American Journal of Public Health*, 107(12):1910–1915, 2017.

[5] Amelia Jamison, David A Broniatowski, Michael C Smith, Kajal S Parikh, Adeena Malik, Mark Dredze, and Sandra C Quinn. Adapting and extending a typology to identify vaccine misinformation on Twitter. *American Journal of Public Health*, 110(S3):S331–S339, 2020.

[6] Christopher A Bail, Taylor W Brown, and Marcus Mann. Channeling hearts and minds: Advocacy organizations, cognitive-emotional currents, and public conversation. *American Sociological Review*, 82(6):1188–1213, 2017.

[7] Hui Yuan, Wei Xu, Qian Li, and Raymond Lau. Topic sentiment mining for sales performance prediction in e-commerce. *Annals of Operations Research*, 270(1):553–576, 2018.

[8] Yingjie Hu, Chengbin Deng, and Zhou Zhou. A semantic and sentiment analysis on online neighborhood reviews for understanding the perceptions of people toward their living environments. *Annals of the American Association of Geographers*, 109(4):1052–1073, 2019.

[9] Yingwei Yan, Jingfu Chen, and Zhiyong Wang. Mining public sentiments and perspectives from geotagged social media data for appraising the post-earthquake recovery of tourism destinations. *Applied Geography*, 123: 102306, 2020.

[10] Babak Hemmatian, Sabina J Sloman, Uriel Cohen Priva, and Steven A Sloman. Think of the consequences: A decade of discourse about same-sex marriage. *Behavior Research Methods*, 51(4):1565–1585, 2019.

[11] Bernd Resch, Florian Usländer, and Clemens Havas. Combining machine-learning topic models and spatiotemporal analysis of social media data for disaster footprint and damage assessment. *Cartography and Geographic Information Science*, 45(4):362–376, 2018.

[12] Georgia Bateman, Hassan Abdel Haleem, and Arnab Majumdar. Is user-generated social media content useful for informing planning and management of emergency events?–An investigation of an active shooting event in a US Airport. *Case Studies on Transport Policy*, 9(3):1015–1025, 2021.

[13] Bangren Zhu, Xinqi Zheng, Haiyan Liu, Jiayang Li, and Peipei Wang. Analysis of spatiotemporal characteristics of big data on social media sentiment with COVID-19 epidemic topics. *Chaos, Solitons & Fractals*, 140: 110123, 2020.

[14] Joyce Y Lee, Olivia D Chang, and Tawfiq Ammari. Using social media Reddit data to examine foster families' concerns and needs during COVID-19. *Child Abuse & Neglect*, 121:105262, 2021.

[15] Mohammed Abdul-Rahman, Edwin HW Chan, Man Sing Wong, Victor E Irekponor, and Maryam O Abdul-Rahman. A framework to simplify pre-processing location-based social media big data for sustainable urban planning and management. *Cities*, 109:102986, 2021.

[16] Gabriela Gongora Svartzman, Jose E Ramirez-Marquez, and Kash Barker. Social media analytics to connect system performability and quality of experience, with an application to Citibike. *Computers & Industrial Engineering*, 139:106146, 2020.

[17] Ahmed Aleroud, Nisreen Abu-Alsheeh, and Emad Al-Shawakfa. A graph proximity feature augmentation approach for identifying accounts of terrorists on Twitter. *Computers & Security*, 99:102056, 2020.

[18] Yihua Su, Aarthi Venkat, Yadush Yadav, Lisa B Puglisi, and Samah J Fodeh. Twitter-based analysis reveals differential COVID-19 concerns across areas with socioeconomic disparities. *Computers in Biology and Medicine*, 132:104336, 2021.

[19] Diana Fischer-Preßler, Carsten Schwemmer, and Kai Fischbach. Collective sense-making in times of crisis: Connecting terror management theory with twitter user reactions to the Berlin terrorist attack. *Computers in Human Behavior*, 100:138–151, 2019.

[20] Noor Farizah Ibrahim and Xiaojun Wang. Decoding the sentiment dynamics of online retailing customers: Time series analysis of social media. *Computers in Human Behavior*, 96:32–45, 2019.

[21] Sifan Xu and Alvin Zhou. Hashtag homophily in Twitter network: Examining a controversial cause-related marketing campaign. *Computers in Human Behavior*, 102:87–96, 2020.

[22] Guy Lansley and Paul A Longley. The geography of Twitter topics in London. *Computers, Environment and Urban Systems*, 58:85–96, 2016.

[23] Cheng Fu, Grant McKenzie, Vanessa Frias-Martinez, and Kathleen Stewart. Identifying spatiotemporal urban activities through linguistic signatures. *Computers, Environment and Urban Systems*, 72:25–37, 2018.

[24] Nicole Marie Brown. Methodological cyborg as black feminist technology: Constructing the social self using computational digital autoethnography and social media. *Cultural Studies ↔ Critical Methodologies*, 19(1):55–67, 2019.

[25] Sebastian Kurten and Kathleen Beullens. #coronavirus: Monitoring the belgian twitter discourse on the severe acute respiratory syndrome coronavirus 2 pandemic. *Cyberpsychology, Behavior, and Social Networking*, 24(2):117–122, 2021.

[26] Noor Farizah Ibrahim and Xiaojun Wang. A text analytics approach for online retailing service improvement: Evidence from Twitter. *Decision Support Systems*, 121:37–50, 2019.

[27] Momin M Malik and Jürgen Pfeffer. A macroscopic analysis of news content in Twitter. *Digital Journalism*, 4(8):955–979, 2016.

[28] Xinzhi Zhang. Visualization, technologies, or the public? Exploring the articulation of data-driven journalism in the Twittersphere. *Digital Journalism*, 6(6):737–758, 2018.

[29] Kjerstin Thorson, Mel Medeiros, Kelley Cotter, Yingying Chen, Kourtnie Rodgers, Arram Bae, and Sevgi Baykaldi. Platform civics: Facebook in the local information infrastructure. *Digital Journalism*, 8(10):1231–1257, 2020.

[30] Andreas Gregoriades and Maria Pampaka. Electronic word of mouth analysis for new product positioning evaluation. *Electronic Commerce Research and Applications*, 42:100986, 2020.

[31] Travis R Meyer, Daniel Balague, Miguel Camacho-Collados, Hao Li, Katie Khuu, P Jeffrey Brantingham, and Andrea L Bertozzi. A year in Madrid as described through the analysis of geotagged Twitter data. *Environment and Planning B: Urban Analytics and City Science*, 46(9):1724–1740, 2019.

[32] Cornelius Puschmann, Julian Ausserhofer, and Josef Šlerka. Converging on a nativist core? Comparing issues on the Facebook pages of the Pegida movement and the alternative for Germany. *European Journal of Communication*, 35(3):230–248, 2020.

[33] Janine Hacker, Jan vom Brocke, Joshua Handali, Markus Otto, and Johannes Schneider. Virtually in this together–how web-conferencing systems enabled a new virtual togetherness during the COVID-19 crisis. *European Journal of Information Systems*, 29(5):563–584, 2020.

[34] Tim K Mackey and Janani Kalyanam. Detection of illicit online sales of fentanyls via Twitter. *F1000Research*, 6, 2017.

[35] Daniel Taninecz Miller. Topics and emotions in Russian Twitter propaganda. *First Monday*, 2019.

[36] Jeff Hemsley, Ingrid Erickson, Mohammad Hossein Jarrahi, and Amir Karami. Digital nomads, coworking, and other expressions of mobile work on Twitter. *First Monday*, 2020.

[37] Maxime Bérubé, Thuc-Uyên Tang, Francis Fortin, Sefa Ozalp, Matthew L Williams, and Pete Burnap. Social media forensics applied to assessment of post–critical incident social reaction: The case of the 2017 Manchester Arena terrorist attack. *Forensic Science International*, 313:110364, 2020.

[38] Yong Li, Mengsi Cai, Shuo Qin, and Xin Lu. Depressive emotion detection and behavior analysis of men who have sex with men via social media. *Frontiers in Psychiatry*, 11:830, 2020.

[39] Colin Klein, Peter Clutton, and Vince Polito. Topic modeling reveals distinct interests within an online conspiracy forum. *Frontiers in Psychology*, 9:189, 2018.

[40] Diogo Nolasco and Jonice Oliveira. Subevents detection through topic modeling in social media posts. *Future Generation Computer Systems*, 93:290–303, 2019.

[41] Oliver Lock and Christopher Pettit. Social media as passive geo-participation in transportation planning–how effective are topic modeling & sentiment analysis in comparison with citizen surveys? *Geo-spatial Information Science*, 23(4):275–292, 2020.

[42] Hong Joo Lee, Minsik Lee, Habin Lee, and Ruth Angelie Cruz. Mining service quality feedback from social media: A computational analytics method. *Government Information Quarterly*, 38(2):101571, 2021.

[43] Alina Pavlova and Pauwke Berkers. “Mental health” as defined by Twitter: Frames, emotions, stigma. *Health Communication*, pages 1–11, 2020.

[44] Volha Murashka, Jiaying Liu, and Yilang Peng. Fitspiration on Instagram: Identifying topic clusters in user comments to posts with objectification features. *Health Communication*, pages 1–12, 2020.

[45] Yang Liu, Christopher Whitfield, Tianyang Zhang, Amanda Hauser, Taeyonn Reynolds, and Mohd Anwar. Monitoring COVID-19 pandemic through the lens of social media using Natural Language Processing and machine learning. *Health Information Science and Systems*, 9(1):1–16, 2021.

[46] Ana Reyes-Menendez, Jose Ramon Saura, and Ferrão Filipe. Marketing challenges in the #MeToo era: Gaining business insights using an exploratory sentiment analysis. *Heliyon*, 6(3):e03626, 2020.

[47] Amalie Dyda, Zubair Shah, Didi Surian, Paige Martin, Enrico Coiera, Aditi Dey, Julie Leask, and Adam G Dunn. HPV vaccine coverage in Australia and associations with HPV vaccine information exposure among Australian Twitter users. *Human Vaccines & Immunotherapeutics*, 15(7-8):1488–1495, 2019.

[48] Namuk Ko, Byeongki Jeong, Sungchul Choi, and Janghyeok Yoon. Identifying product opportunities using social media mining: Application of topic modeling and chance discovery theory. *IEEE Access*, 6:1680–1693, 2018. doi: 10.1109/ACCESS.2017.2780046.

[49] José Tomás Méndez, Hans Lobel, Denis Parra, and Juan Carlos Herrera. Using Twitter to infer user satisfaction with public transport: the case of Santiago, Chile. *IEEE Access*, 7:60255–60263, 2019.

[50] Mohammed Bahja and Ghazanfar Ali Safdar. Unlink the link between COVID-19 and 5G networks: an NLP and SNA based approach. *IEEE Access*, 8:209127–209137, 2020.

[51] Leonardo Nizzoli, Serena Tardelli, Marco Avvenuti, Stefano Cresci, Maurizio Tesconi, and Emilio Ferrara. Charting the landscape of online cryptocurrency manipulation. *IEEE Access*, 8:113230–113245, 2020.

[52] Xia Liu. Analyzing the impact of user-generated content on firms' stock performance: Big data analysis with machine learning methods. *Industrial Marketing Management*, 86:30–39, 2020.

[53] Sameh N Saleh, Christoph U Lehmann, Samuel A McDonald, Mujeeb A Basit, and Richard J Medford. Understanding public perception of Coronavirus disease 2019 (COVID-19) social distancing on twitter. *Infection Control & Hospital Epidemiology*, 42(2):131–138, 2021.

[54] Tobias Brandt, Johannes Bendler, and Dirk Neumann. Social media analytics and value creation in urban smart tourism ecosystems. *Information & Management*, 54(6):703–713, 2017.

[55] Sejung Park, Dahoon Chung, and Han Woo Park. Analytical framework for evaluating digital diplomacy using network analysis and topic modeling: Comparing South Korea and Japan. *Information Processing & Management*, 56(4):1468–1483, 2019.

[56] Mike Thelwall and Emma Stuart. She's Reddit: A source of statistically significant gendered interest information? *Information processing & management*, 56(4):1543–1558, 2019.

[57] Adewale Obadimu, Tuja Khaund, Esther Mead, Thomas Marcoux, and Nitin Agarwal. Developing a socio-computational approach to examine toxicity propagation and regulation in COVID-19 discourse on YouTube. *Information Processing & Management*, 58(5):102660, 2021.

[58] Patrick Cheong-Iao Pang, Dana McKay, Shanton Chang, Qingyu Chen, Xiuzhen Zhang, and Lishan Cui. Privacy concerns of the Australian My Health Record: Implications for other large-scale opt-out personal health records. *Information Processing & Management*, 57(6):102364, 2020.

[59] Arpan Kumar Kar. What affects usage satisfaction in mobile payments? Modelling user generated content to develop the "digital service usage satisfaction model". *Information Systems Frontiers*, 23(5):1341–1361, 2021.

[60] Tian Yang and Kecheng Fang. How dark corners collude: A study on an online Chinese alt-right community. *Information, Communication & Society*, pages 1–18, 2021.

[61] Sebastian Stier, Lisa Posch, Arnim Bleier, and Markus Strohmaier. When populists become popular: comparing Facebook use by the right-wing movement Pegida and German political parties. *Information, Communication & Society*, 20(9):1365–1388, 2017.

[62] Naomi Smith and Tim Graham. Mapping the anti-vaccination movement on Facebook. *Information, Communication & Society*, 22(9):1310–1327, 2019.

[63] Pei Zheng and Saif Shahin. Live tweeting live debates: How Twitter reflects and refracts the US political climate in a campaign season. *Information, Communication & Society*, 23(3):337–357, 2020.

[64] Linwan Wu, Naa Amponsah Dodoo, Taylor Jing Wen, and Li Ke. Understanding Twitter conversations about artificial intelligence in advertising based on natural language processing. *International Journal of Advertising*, pages 1–18, 2021.

[65] K Hazel Kwon, Monica Chadha, and Feng Wang. Proximity and networked news public: Structural topic modeling of global Twitter conversations about the 2017 Quebec mosque shooting. *International Journal of Communication*, 13:2652–2675, 2019.

[66] Shiwen Wu and Bo Mai. Talking about and beyond censorship: Mapping topic clusters in the Chinese Twitter sphere. *International Journal of Communication*, 13:23, 2019.

[67] Muhammed Ali Sit, Caglar Koylu, and Ibrahim Demir. Identifying disaster-related tweets and their semantic, spatial and temporal context using deep learning, Natural Language Processing and spatial analysis: a case study of Hurricane Irma. *International Journal of Digital Earth*, 2019.

[68] Qing Deng, Yang Gao, Chenyang Wang, and Hui Zhang. Detecting information requirements for crisis communication from social media data: An interactive topic modeling approach. *International Journal of Disaster Risk Reduction*, 50:101692, 2020.

[69] Wei Zhai, Zhong-Ren Peng, and Faxi Yuan. Examine the effects of neighborhood equity on disaster situational awareness: Harness machine learning and geotagged Twitter data. *International Journal of Disaster Risk Reduction*, 48:101611, 2020.

[70] Wenjun Wu, Junli Li, Zongyi He, Xinxin Ye, Jie Zhang, Xiu Cao, and Hongjiao Qu. Tracking spatio-temporal variation of geo-tagged topics with social media in China: A case study of 2016 Hefei rainstorm. *International Journal of Disaster Risk Reduction*, 50:101737, 2020.

[71] Faxi Yuan, Min Li, and Rui Liu. Understanding the evolutions of public responses using social media: Hurricane Matthew case study. *International Journal of Disaster Risk Reduction*, 51:101798, 2020.

[72] Katsushige Kitazawa and Scott A Hale. Social media and early warning systems for natural disasters: A case study of Typhoon Etau in Japan. *International Journal of Disaster Risk Reduction*, 52:101926, 2021.

[73] Markus Moessner, Johannes Feldhege, Markus Wolf, and Stephanie Bauer. Analyzing big data in social media: Text and network analyses of an eating disorder forum. *International Journal of Eating Disorders*, 51(7):656–667, 2018.

[74] Wen Deng and Yi Yang. Cross-platform comparative study of public concern on social media during the COVID-19 pandemic: An empirical study based on Twitter and Weibo. *International Journal of Environmental Research and Public Health*, 18(12):6487, 2021.

[75] Amir Karami, Morgan Lundy, Frank Webb, Gabrielle Turner-McGrievy, Brooke W McKeever, and Robert McKeever. Identifying and analyzing health-related themes in disinformation shared by conservative and liberal Russian trolls on Twitter. *International Journal of Environmental Research and Public Health*, 18(4):2159, 2021.

[76] Yanan Xin and Alan M MacEachren. Characterizing traveling fans: a workflow for event-oriented travel pattern analysis using Twitter data. *International Journal of Geographical Information Science*, 34(12):2497–2516, 2020.

[77] Ting Zhang, Shi Shen, Changxiu Cheng, Kai Su, and Xiangxue Zhang. A topic model based framework for identifying the distribution of demand for relief supplies using social media data. *International Journal of Geographical Information Science*, pages 1–22, 2021.

[78] Amir Karami, Alicia A Dahl, Gabrielle Turner-McGrievy, Hadi Kharrazi, and George Shaw Jr. Characterizing diabetes, diet, exercise, and obesity comments on twitter. *International Journal of Information Management*, 38(1):1–6, 2018.

[79] Jaebong Son, Hyung Koo Lee, Sung Jin, and Jintae Lee. Content features of tweets for effective communication during disasters: A media synchronicity theory perspective. *International Journal of Information Management*, 45:56–68, 2019.

[80] Byeongki Jeong, Janghyeok Yoon, and Jae-Min Lee. Social media mining for product planning: A product opportunity mining approach based on topic modeling and sentiment analysis. *International Journal of Information Management*, 48:280–290, 2019.

[81] Byeongki Jeong, Janghyeok Yoon, and Jae-Min Lee. Social media mining for product planning: A product opportunity mining approach based on topic modeling and sentiment analysis. *International Journal of Information Management*, 48:280–290, 2019.

[82] Diogo Nolasco and Jonice Oliveira. Mining social influence in science and vice-versa: A topic correlation approach. *International Journal of Information Management*, 51:102017, 2020.

[83] Faxi Yuan, Min Li, Rui Liu, Wei Zhai, and Bing Qi. Social media for enhanced understanding of disaster resilience during Hurricane Florence. *International Journal of Information Management*, 57:102289, 2021.

[84] Ling Zhang, Magie Hall, and Dhundy Bastola. Utilizing Twitter data for analysis of chemotherapy. *International Journal of Medical Informatics*, 120:92–100, 2018.

[85] Jia-Wen Guo, Shawna M Sisler, Ching-Yu Wang, and Andrea S Wallace. Exploring experiences of COVID-19-positive individuals from social media posts. *International Journal of Nursing Practice*, 27(5):e12986, 2021.

[86] Raphael Heiberger, Silvia Majó-Vázquez, Laia Castro Herrero, Rasmus K Nielsen, and Frank Esser. Do Not Blame the Media! the role of politicians and parties in fragmenting online political debate. *The International Journal of Press/Politics*, 2021.

[87] Qi Gao, Fabian Abel, Geert-Jan Houben, and Yong Yu. A comparative study of users' microblogging behavior on Sina Weibo and Twitter. In *International Conference on User Modeling, Adaptation, and Personalization*, pages 88–101. Springer, 2012.

[88] Zhan Xu, Kenneth Lachlan, Lauren Ellis, and Adam Michael Rainear. Understanding public opinion in different disaster stages: A case study of Hurricane Irma. *Internet Research*, 30(2):695–709, 2019.

[89] Nabila El-Bassel, Karli R Hochstatter, Melissa N Slavin, Chenghao Yang, Yudong Zhang, and Smaranda Muresan. Harnessing the power of social media to understand the impact of COVID-19 on people who use drugs during lockdown and social distancing. *Journal of Addiction Medicine*, 2021.

[90] Emanuele Fino, Bishoy Hanna-Khalil, and Mark D Griffiths. Exploring the public's perception of gambling addiction on Twitter during the COVID-19 pandemic: Topic modelling and sentiment analysis. *Journal of Addictive Diseases*, 39(4):489–503, 2021.

[91] Xia Liu, Alvin C Burns, and Yingjian Hou. An investigation of brand-related user-generated content on Twitter. *Journal of Advertising*, 46(2): 236–247, 2017.

[92] Johannes Feldhege, Markus Moessner, and Stephanie Bauer. Who says what? Content and participation characteristics in an online depression community. *Journal of Affective Disorders*, 263:521–527, 2020.

[93] Lixia Yu, Wanyue Jiang, Zhihong Ren, Sheng Xu, Lin Zhang, and Xiangen Hu. Detecting changes in attitudes toward depression on Chinese social media: A text analysis. *Journal of Affective Disorders*, 280:354–363, 2021.

[94] Charlene Zhang, Martin C Yu, and Sebastian Marin. Exploring public sentiment on enforced remote work during COVID-19. *Journal of Applied Psychology*, 106(6):797, 2021.

[95] Julia Wu, Venkatesh Sivaraman, Dheekshita Kumar, Juan M Banda, and David Sontag. Pulse of the pandemic: Iterative topic filtering for clinical information extraction from social media. *Journal of Biomedical Informatics*, 120:103844, 2021.

[96] Ying Wang, Heng Li, and Zezhou Wu. Attitude of the Chinese public toward off-site construction: A text mining study. *Journal of Cleaner Production*, 238:117926, 2019.

[97] Vinay Kumar Jain and Shishir Kumar. Effective surveillance and predictive mapping of mosquito-borne diseases using social media. *Journal of Computational Science*, 25:406–415, 2018.

[98] Samuel Merrill and Mathilda Åkerlund. Standing up for sweden? The racist discourses, architectures and affordances of an anti-immigration Facebook group. *Journal of Computer-Mediated Communication*, 23(6): 332–353, 2018.

[99] Anish K Agarwal, Vivien Wong, Arthur M Pelullo, Guntuku Sharath, Daniel Polsky, David A Asch, Muruako Jonathan, and Raina M Merchant. Online reviews of specialized drug treatment facilities—identifying potential drivers of high and low patient satisfaction. *Journal of General Internal Medicine*, 35(6):1647–1653, 2020.

[100] Daniel C Stokes, Jonathan Purtle, Zachary F Meisel, and Anish K Agarwal. State legislators' divergent social media response to the opioid epidemic from 2014 to 2019: longitudinal topic modeling analysis. *Journal of General Internal Medicine*, 36(11):3373–3382, 2021.

[101] Bo Liang, Ye Wang, and Ming-Hsiang Tsou. A "fitness" theme may mitigate regional prevalence of overweight and obesity: Evidence from Google search and tweets. *Journal of Health Communication*, 24(9):683–692, 2019.

[102] Chad A Melton, Olufunto A Olusanya, Nariman Ammar, and Arash Shaban-Nejad. Public sentiment analysis and topic modeling regarding COVID-19 vaccines on the Reddit social media platform: A call to action for strengthening vaccine confidence. *Journal of Infection and Public Health*, 14(10):1505–1512, 2021.

[103] Amir Karami, Vishal Shah, Reza Vaezi, and Amit Bansal. Twitter speaks: A case of national disaster situational awareness. *Journal of Information Science*, 46(3):313–324, 2020.

[104] Sulong Zhou, Pengyu Kan, Qunying Huang, and Janet Silbernagel. A guided Latent Dirichlet Allocation approach to investigate real-time latent topics of Twitter data during Hurricane Laura. *Journal of Information Science*, 2021.

[105] Michael Vaughan. Talking about tax: the discursive distance between 38 Degrees and GetUp. *Journal of Information Technology & Politics*, 17(2): 114–129, 2020.

[106] Man-pui Sally Chan, Kathleen Hall Jamieson, and Dolores Albarracin. Prospective associations of regional social media messages with attitudes and actual vaccination: A big data and survey study of the influenza vaccine in the United States. *Vaccine*, 38(40):6236–6247, 2020.

[107] Bing Qi, Aaron Costin, and Mengda Jia. A framework with efficient extraction and analysis of Twitter data for evaluating public opinions on transportation services. *Travel Behaviour and Society*, 21:10–23, 2020.

[108] Andrei P Kirilenko, Svetlana O Stepchenkova, and Xiangyi Dai. Automated topic modeling of tourist reviews: Does the Anna Karenina principle apply? *Tourism Management*, 83:104241, 2021.

[109] Rong Wang and Alvin Zhou. Hashtag activism and connective action: A case study of#hongkongpolicebrutality. *Telematics and Informatics*, 61: 101600, 2021.

[110] Marlen Komorowski, Tien Do Huu, and Nikos Deligiannis. Twitter data analysis for studying communities of practice in the media industry. *Telematics and Informatics*, 35(1):195–212, 2018.

[111] Mohamed M Mostafa and Nicolás Roser Nebot. The Arab image in Spanish social media: A Twitter sentiment analytics approach. *Journal of Intercultural Communication Research*, 49(2):133–155, 2020.

[112] Renana Peres, Sunali Talwar, Liav Alter, Michal Elhanan, and Yuval Friedmann. Narrowband influencers and global icons: Universality and media compatibility in the communication patterns of political leaders worldwide. *Journal of International Marketing*, 28(1):48–65, 2020.

[113] Taehyun Ha, Bjorn Beijnon, Sangyeon Kim, Sangwon Lee, and Jang Hyun Kim. Examining user perceptions of smartwatch through dynamic topic modeling. *Telematics and Informatics*, 34(7):1262–1273, 2017.

[114] Bongsug Kevin Chae. The evolution of the Internet of Things (IoT): A computational text analysis. *Telecommunications Policy*, 43(10):101848, 2019.

[115] Neal Shah, Jiawei Li, and Tim K Mackey. An unsupervised machine learning approach for the detection and characterization of illicit drug-dealing comments and interactions on Instagram. *Substance Abuse*, pages 1–5, 2021.

[116] Pengxiang Li, Hichang Cho, Yuren Qin, and Anfan Chen. #MeToo as a connective movement: Examining the frames adopted in the anti-sexual harassment movement in China. *Social Science Computer Review*, 39(5): 1030–1049, 2020.

[117] Alina Pavlova and Pauwke Berkers. Mental health discourse and social media: Which mechanisms of cultural power drive discourse on Twitter. *Social Science & Medicine*, 263:113250, 2020.

[118] Christopher A Bail. Cultural carrying capacity: Organ donation advocacy, discursive framing, and social media engagement. *Social Science & Medicine*, 165:280–288, 2016.

[119] Ikechukwu Onyenwe, Samuel Nwagbo, Njideka Mbeledogu, and Ebele Onyedinma. The impact of political party/candidate on the election results from a sentiment analysis perspective using #AnambraDecides2017 tweets. *Social Network Analysis and Mining*, 10(1):1–17, 2020.

[120] Biraj Dahal, Sathish AP Kumar, and Zhenlong Li. Topic modeling and sentiment analysis of global climate change tweets. *Social Network Analysis and Mining*, 9(1):1–20, 2019.

[121] Supraja Gurajala, Suresh Dhaniyala, and Jeanna N Matthews. Understanding public response to air quality using tweet analysis. *Social Media + Society*, 5(3):1–14, 2019.

[122] Patrick Rafail and Isaac Freitas. Grievance articulation and community reactions in the men's rights movement online. *Social Media + Society*, 5 (2), 2019.

[123] Emiliano del Gobbo, Sara Fontanella, Annalina Sarra, and Lara Fontanella. Emerging topics in Brexit debate on Twitter around the deadlines. *Social Indicators Research*, 156(2):669–688, 2021.

[124] Mi Kyung Lee, Ho Young Yoon, Marc Smith, Hye Jin Park, and Han Woo Park. Mapping a Twitter scholarly communication network: a case of the association of internet researchers' conference. *Scientometrics*, 112(2): 767–797, 2017.

[125] Zezhou Wu, Yan Zhang, Qiaohui Chen, and Hao Wang. Attitude of Chinese public towards municipal solid waste sorting policy: A text mining study. *Science of The Total Environment*, 756:142674, 2021.

[126] N Nima Haghighi, Xiaoyue Cathy Liu, Ran Wei, Wenwen Li, and Hu Shao. Using Twitter data for transit performance assessment: a framework for evaluating transit riders' opinions about quality of service. *Public Transport*, 10(2):363–377, 2018.

[127] Sifan Xu and Ying Xiong. Setting socially mediated engagement parameters: A topic modeling and text analytic approach to examining polarized discourses on Gillette's campaign. *Public Relations Review*, 46(5):101959, 2020.

[128] LiYaning Tang, Yiming Zhang, Fei Dai, Yoojung Yoon, Yangqiu Song, and Radhey S Sharma. Social media data analytics for the US construction industry: Preliminary study on Twitter. *Journal of Management in Engineering*, 33(6):04017038, 2017.

[129] Didi Surian, Dat Quoc Nguyen, Georgina Kennedy, Mark Johnson, Enrico Coiera, and Adam G Dunn. Characterizing Twitter discussions about HPV vaccines using topic modeling and community detection. *Journal of Medical Internet Research*, 18(8):e6045, 2016.

[130] Ireneus Kagashe, Zhijun Yan, and Imran Suheryani. Enhancing seasonal influenza surveillance: topic analysis of widely used medicinal drugs using Twitter data. *Journal of Medical Internet Research*, 19(9):e315, 2017.

[131] Gisele Lobo Pappa, Tiago Oliveira Cunha, Paulo Viana Bicalho, Antonio Ribeiro, Ana Paula Couto Silva, Wagner Meira Jr, and Alline Maria Rezende Beleigoli. Factors associated with weight change in online weight management communities: a case study in the LoseIt Reddit community. *Journal of Medical Internet Research*, 19(1):e17, 2017.

[132] Xinyan Zhao, Mengqi Zhan, and Cheng Jie. Examining multiplicity and dynamics of publics' crisis narratives with large-scale Twitter data. *Public Relations Review*, 44(4):619–632, 2018.

[133] Sejung Park and Han Woo Park. A webometric network analysis of electronic word of mouth (eWOM) characteristics and machine learning approach to consumer comments during a crisis. *EL Profesional De La Información*, 29(5), 2020.

[134] Kevin Aslett, Nora Webb Williams, Andreu Casas, Wesley Zuidema, and John Wilkerson. What was the problem in Parkland? Using social media to measure the effectiveness of issue frames. *Policy Studies Journal*, 2020.

[135] Sara Moukarzel, Martin Rehm, Anita Caduff, Miguel Del Fresno, Rafael Perez-Escamilla, and Alan J Daly. Real-time Twitter interactions during World Breastfeeding Week: A case study and social network analysis. *PloS One*, 16(3):e0249302, 2021.

[136] Mingxiang Cai, Neal Shah, Jiawei Li, Wen-Hao Chen, Raphael E Cuomo, Nick Obradovich, and Tim K Mackey. Identification and characterization of tweets related to the 2015 Indiana HIV outbreak: A retrospective infoveillance study. *Plos one*, 15(8):e0235150, 2020.

[137] Jia Xue, Junxiang Chen, Chen Chen, Chengda Zheng, Sijia Li, and Tingshao Zhu. Public discourse and sentiment during the COVID-19 pandemic: Using Latent Dirichlet Allocation for topic modeling on Twitter. *PloS one*, 15(9):e0239441, 2020.

[138] Philipp Wicke and Marianna M Bolognesi. Framing COVID-19: How we conceptualize and discuss the pandemic on Twitter. *PloS one*, 15(9): e0240010, 2020.

[139] Dasha Pruss, Yoshinari Fujinuma, Ashlynn R Daughton, Michael J Paul, Brad Arnot, Danielle Albers Szafir, and Jordan Boyd-Graber. Zika discourse in the Americas: A multilingual topic analysis of Twitter. *PloS one*, 14(5):e0216922, 2019.

[140] Gem M Le, Kate Radcliffe, Courtney Lyles, Helena C Lyson, Byron Wallace, George Sawaya, Rena Pasick, Damon Centola, and Urmimala Sarkar. Perceptions of cervical cancer prevention on Twitter uncovered by different sampling strategies. *PloS One*, 14(2):e0211931, 2019.

[141] Jiang Bian, Kenji Yoshigoe, Amanda Hicks, Jiawei Yuan, Zhe He, Mengjun Xie, Yi Guo, Mattia Prosperi, Ramzi Salloum, and François Modave. Mining Twitter to assess the public perception of the "Internet of Things". *PloS One*, 11(7):e0158450, 2016.

[142] Yongcheng Zhan, Ruoran Liu, Qiudan Li, Scott James Leischow, Daniel Dajun Zeng, et al. Identifying topics for e-cigarette user-generated contents: a case study from multiple social media platforms. *Journal of Medical Internet Research*, 19(1):e5780, 2017.

[143] Jiang Bian, Yunpeng Zhao, Ramzi G Salloum, Yi Guo, Mo Wang, Mattia Prosperi, Hansi Zhang, Xinsong Du, Laura J Ramirez-Diaz, Zhe He, et al. Using social media data to understand the impact of promotional information on laypeople's discussions: a case study of lynch syndrome. *Journal of Medical Internet Research*, 19(12):e9266, 2017.

[144] Robert J Smith, Patrick Crutchley, H Andrew Schwartz, Lyle Ungar, Frances Shofer, Kevin A Padrez, and Raina M Merchant. Variations in Facebook posting patterns across validated patient health conditions: a prospective cohort study. *Journal of Medical Internet Research*, 19(1):e7, 2017.

[145] Tim Mackey, Janani Kalyanam, Josh Klugman, Ella Kuzmenko, and Rashmi Gupta. Solution to detect, classify, and report illicit online marketing and sales of controlled substances via Twitter: Using machine learning and web forensics to combat digital opioid access. *Journal of Medical Internet Research*, 20(4):e10029, 2018.

[146] Junze Wang, Ying Zhou, Wei Zhang, Richard Evans, Chengyan Zhu, et al. Concerns expressed by Chinese social media users during the COVID-19 pandemic: content analysis of Sina Weibo microblogging data. *Journal of Medical Internet Research*, 22(11):e22152, 2020.

[147] Raghad Alshalan, Hend Al-Khalifa, Duaa Alsaeed, Heyam Al-Baity, and Shahad Alshalan. Detection of hate speech in COVID-19–related tweets in the Arab region: Deep learning and topic modeling approach. *Journal of Medical Internet Research*, 22(12):e22609, 2020.

[148] Caitlin Doogan, Wray Buntine, Henry Linger, and Samantha Brunt. Public perceptions and attitudes toward COVID-19 nonpharmaceutical interventions across six countries: A topic modeling analysis of Twitter data. *Journal of Medical Internet Research*, 22(9):e21419, 2020.

[149] Danny Valdez, Marijn Ten Thij, Krishna Bathina, Lauren A Rutter, and Johan Bollen. Social media insights into US mental health during the COVID-19 pandemic: Longitudinal analysis of Twitter data. *Journal of Medical Internet Research*, 22(12):e21418, 2020.

[150] Jia Xue, Junxiang Chen, Chen Chen, Ran Hu, and Tingshao Zhu. The hidden pandemic of family violence during COVID-19: Unsupervised learning of tweets. *Journal of Medical Internet Research*, 22(11):e24361, 2020.

[151] Alaa Abd-Alrazaq, Dari Alhuwail, Mowafa Househ, Mounir Hamdi, Zubair Shah, et al. Top concerns of tweeters during the COVID-19 pandemic: Infoveillance study. *Journal of Medical Internet Research*, 22(4): e19016, 2020.

[152] Ranganathan Chandrasekaran, Vikalp Mehta, Tejali Valkunde, and Evangelos Moustakas. Topics, trends, and sentiments of tweets about the COVID-19 pandemic: Temporal infoveillance study. *Journal of Medical Internet Research*, 22(10):e22624, 2020.

[153] Jia Xue, Junxiang Chen, Ran Hu, Chen Chen, Chengda Zheng, Yue Su, and Tingshao Zhu. Twitter discussions and emotions about the COVID-19 pandemic: Machine learning approach. *Journal of Medical Internet Research*, 22(11):e20550, 2020.

[154] Johannes Feldhege, Markus Moessner, Markus Wolf, Stephanie Bauer, et al. Changes in language style and topics in an online eating disorder community at the beginning of the COVID-19 pandemic: Observational study. *Journal of Medical Internet Research*, 23(7):e28346, 2021.

[155] Xinyu Zhou, Yi Song, Hao Jiang, Qian Wang, Zhiqiang Qu, Xiaoyu Zhou, Mark Jit, Zhiyuan Hou, and Leesa Lin. Comparison of public responses to containment measures during the initial outbreak and resurgence of COVID-19 in China: Infodemiology study. *Journal of Medical Internet Research*, 23(4):e26518, 2021.

[156] Sungkyu Park, Sungwon Han, Jeongwook Kim, Mir Majid Molaie, Hoang Dieu Vu, Karandeep Singh, Jiyoung Han, Wonjae Lee, and Meeyoung Cha. COVID-19 discourse on twitter in four Asian countries: Case study of risk communication. *Journal of Medical Internet Research*, 23 (3):e23272, 2021.

[157] Siru Liu, Jili Li, Jialin Liu, et al. Leveraging transfer learning to analyze opinions, attitudes, and behavioral intentions toward COVID-19 vaccines: Social media content and temporal analysis. *Journal of Medical Internet Research*, 23(8):e30251, 2021.

[158] Chengda Zheng, Jia Xue, Yumin Sun, and Tingshao Zhu. Public opinions and concerns regarding the Canadian Prime Minister's daily COVID-19 briefing: Longitudinal study of Youtube comments using machine learning techniques. *Journal of Medical Internet Research*, 23(2):e23957, 2021.

[159] Colton Margus, Natasha Brown, Attila J Hertelendy, Michelle R Safferman, Alexander Hart, Gregory R Ciottone, et al. Emergency physician

Twitter use in the COVID-19 pandemic as a potential predictor of impending surge: Retrospective observational study. *Journal of Medical Internet Research*, 23(7):e28615, 2021.

[160] Hyeju Jang, Emily Rempel, David Roth, Giuseppe Carenini, and Naveed Zafar Janjua. Tracking COVID-19 discourse on Twitter in North America: Infodemiology study using topic modeling and aspect-based sentiment analysis. *Journal of Medical Internet Research*, 23(2):e25431, 2021.

[161] Stephen Wai Hang Kwok, Sai Kumar Vadde, and Guanjin Wang. Tweet topics and sentiments relating to COVID-19 vaccination among Australian Twitter users: Machine learning analysis. *Journal of Medical Internet Research*, 23(5):e26953, 2021.

[162] Jörg Haßler, Anna-Katharina Wurst, Marc Jungblut, and Katharina Schlosser. Influence of the pandemic lockdown on Fridays for Future's hashtag activism. *New Media & Society*, 2021.

[163] Youjin Hwang, Hyung Jun Kim, Hyung Jin Choi, and Joonhwan Lee. Exploring abnormal behavior patterns of online users with emotional eating behavior: topic modeling study. *Journal of Medical Internet Research*, 22 (3):e15700, 2020.

[164] Andrew Jenkins, Arie Croitoru, Andrew T Crooks, and Anthony Stefanidis. Crowdsourcing a collective sense of place. *PloS One*, 11(4):e0152932, 2016.

[165] Jedidiah Carlson and Kelley Harris. Quantifying and contextualizing the impact of bioRxiv preprints through automated social media audience segmentation. *PLoS Biology*, 18(9):e3000860, 2020.

[166] Keke Hou, Tingting Hou, and Lili Cai. Public attention about COVID-19 on social media: An investigation based on data mining and text analysis. *Personality and Individual Differences*, 175:110701, 2021.

[167] Nina Cesare, Olubusola Oladeji, Kadija Ferryman, Derry Wijaya, Karen D Hendricks-Muñoz, Alyssa Ward, and Elaine O Nsoesie. Discussions of miscarriage and preterm births on Twitter. *Paediatric and perinatal epidemiology*, 34(5):544–552, 2020.

[168] Colin Robertson and Lauren Yee. Avian influenza risk surveillance in North America with online media. *PloS One*, 11(11):e0165688, 2016.

[169] Richard J Medford, Sameh N Saleh, Andrew Sumarsono, Trish M Perl, and Christoph U Lehmann. An "infodemic": Leveraging high-volume Twitter data to understand early public sentiment for the Coronavirus disease 2019 outbreak. *Open forum infectious diseases*, 7(7), 2020.

[170] Lu An, Chuanming Yu, Xia Lin, Tingyao Du, Liqin Zhou, and Gang Li. Topical evolution patterns and temporal trends of microblogs on public health emergencies: An exploratory study of Ebola on Twitter and Weibo. *Online information review*, 43(6), 2018.

[171] Marian H Amin, Ehab KA Mohamed, and Ahmed Elragal. Corporate disclosure via social media: a data science approach. *Online Information Review*, 44(1), 2020.

[172] Soohyung Joo, Kun Lu, and Taehun Lee. Analysis of content topics, user engagement and library factors in public library social media based on text mining. *Online information review*, 44(1), 2020.

[173] Yanfen Zhou and Jin-Cheon Na. A comparative analysis of Twitter users who tweeted on psychology and political science journal articles. *Online Information Review*, 43(7):1188–1208, 2019.

[174] Aqdas Malik, Muhammad Irfan Khan, Habib Karbasian, Marko Nieminen, Muhammad Ammad-Ud-Din, and Suleiman Ali Khan. Modeling public sentiments about JUUL flavors on Twitter through machine learning. *Nicotine and Tobacco Research*, 23(11):1869–1879, 2021.

[175] Simin Kargar and Adrian Rauchfleisch. State-aligned trolling in iran and the double-edged affordances of Instagram. *New media & Society*, 21(7): 1506–1527, 2019.

[176] Sebastian Berg, Tim König, and Ann-Kathrin Koster. Political opinion formation as epistemic practice: The hashtag assemblage of# metwo. *Media and Communication*, 8(4):84–95, 2020.

[177] P Otero, J Gago, and P Quintas. Twitter data analysis to assess the interest of citizens on the impact of marine plastic pollution. *Marine Pollution Bulletin*, 170:112620, 2021.

[178] Xiaoling Xiang, Xuan Lu, Alex Halavanau, Jia Xue, Yihang Sun, Patrick Ho Lam Lai, and Zhenke Wu. Modern senicide in the face of a pandemic: An examination of public discourse and sentiment about older adults and COVID-19 using machine learning. *The Journals of Gerontology: Series B*, 76(4):e190–e200, 2021.

[179] Hansi Zhang, Christopher Wheldon, Adam G Dunn, Cui Tao, Jinhai Huo, Rui Zhang, Mattia Prosperi, Yi Guo, and Jiang Bian. Mining Twitter to assess the determinants of health behavior toward Human Papillomavirus vaccination in the United States. *Journal of the American Medical Informatics Association*, 27(2):225–235, 2020.

[180] Edidiong Okon, Vishnutheja Rachakonda, Hyo Jung Hong, Chris Callison-Burch, and Jules B Lipoff. Natural Language Processing of Reddit data to evaluate dermatology patient experiences and therapeutics. *Journal of the American Academy of Dermatology*, 83(3):803–808, 2020.

[181] Xia Liu. A big data approach to examining social bots on Twitter. *Journal of Services Marketing*, 2019.

[182] Jia-Wen Guo, Djin Lyn Tay, and Michelle L Litchman. Hashtags and heroes: perceptions of nursing on Twitter following a high profile nurse arrest. *Journal of Professional Nursing*, 35(5):398–404, 2019.

[183] Daniel J Smith, Valerie VT Mac, and Vicki S Hertzberg. Using twitter for nursing research: A tweet analysis on heat illness and health. *Journal of Nursing Scholarship*, 53(3):343–350, 2021.

[184] Maurizio Massaro, Paola Tamburro, Matteo La Torre, Francesca Dal Mas, Ronald Thomas, Lorenzo Cobianchi, and Paul Barach. Non-pharmaceutical interventions and the infodemic on Twitter: Lessons learned from Italy during the COVID-19 pandemic. *Journal of Medical Systems*, 45(4):1–12, 2021.

[185] Yang Liu, Zhijun Yin, et al. Understanding weight loss via online discussions: Content analysis of Reddit posts using topic modeling and word clustering techniques. *Journal of Medical Internet Research*, 22(6):e13745, 2020.

[186] Joanne Chen Lyu and Garving K Luli. Understanding the public discussion about the Centers for Disease Control and prevention during the COVID-19 pandemic using Twitter data: Text mining analysis study. *Journal of Medical Internet Research*, 23(2):e25108, 2021.

[187] Colton Margus, Natasha Brown, Attila Hertelendy, Michelle R Safferman, Alexander Hart, and Gregory R Ciottone. Social Media and the Surge: Emergency physician Twitter use in the COVID-19 pandemic as a potential predictor of impending surge. *Journal of Medical Internet Research*, 2021.

[188] Daniel M Low, Laurie Rumker, Tanya Talkar, John Torous, Guillermo Cecchi, and Satrajit S Ghosh. Natural Language Processing reveals vulnerable mental health support groups and heightened health anxiety on Reddit during COVID-19: Observational study. *Journal of Medical Internet Research*, 22(10):e22635, 2020.

[189] Long Chen, Xinyi Lu, Jianbo Yuan, Joyce Luo, Jiebo Luo, Zidian Xie, and Dongmei Li. A social media study on the associations of flavored electronic cigarettes with health symptoms: Observational study. *Journal of Medical Internet Research*, 22(6):e17496, 2020.

[190] SWH Kwok, SK Vadde, and G Wang. Twitter speaks: An analysis of Australian Twitter users' topics and sentiments about COVID-19 vaccination using machine learning. *Journal of Medical Internet Research*, 2021.

[191] Wenhao Chen, Kin Keung Lai, and Yi Cai. Exploring public mood toward commodity markets: a comparative study of user behavior on Sina Weibo and Twitter. *Internet Research*, 2020.
